# Herbal medicines in Alzheimer’s disease and the involvement of gut microbiota

**DOI:** 10.3389/fphar.2024.1416502

**Published:** 2024-07-16

**Authors:** Mingli Liu, Tuming Li, Huazheng Liang, Ping Zhong

**Affiliations:** ^1^ Department of Neurology, Yangpu District Shidong Hospital Affiliated to University of Shanghai for Science and Technology, Shanghai, China; ^2^ Shanghai Key Laboratory of Anesthesiology and Brain Functional Modulation, Clinical Research Center for Anesthesiology and Perioperative Medicine, Shanghai Fourth People’s Hospital, School of Medicine, Tongji University, Shanghai, China; ^3^ Monash Suzhou Research Institute, Suzhou, China

**Keywords:** Alzheimer’s disease, gut microbiota, herbal medicine, herbal formulae, therapeutic targets

## Abstract

Alzheimer’s disease (AD) is a progressive neurodegenerative disorder characterized by memory loss and cognitive impairment. It severely affects the quality of life of victims. The prevalence of AD has been increasing in recent years. Therefore, it is of great importance to elucidate the pathogenic mechanism of AD and search for effective therapeutic approaches. Gut microbiota dysbiosis, an altered state of gut microbiota, has been well known for its involvement in the pathogenesis of AD. Much effort has been made in searching for approaches capable of modulating the composition of gut microbiota in recent years. Herbal medicines have attracted extensive attention in recent decades for the prevention and treatment of AD. Here, we gave an overview of the recent research progress on the modulatory effects of herbal medicines and herbal formulae on gut microbiota as well as the possible beneficial effects on AD, which may provide new insights into the discovery of anti-AD agents and their therapeutic potential for AD through modulating the composition of gut microbiota.

## 1 Introduction

Alzheimer’s disease, one of the leading causes of morbidity, disability, and death in the elderly, is an irreversible neurodegenerative condition. It is estimated that a large number of elderly people are living with AD nowadays and the prevalence and incidence of AD is continuing to grow as the population ages (Cui et al., 2020; ALZHEIMER S ASSOCIATION REPORT, 2021). This brings a heavy burden to both their families and the healthcare system (Agudelo-Botero et al., 2023). AD is characterized by specific pathological hallmarks, including extracellular amyloid β (Aβ) plaques (Paroni et al., 2019), intracellular neurofibrillary tangles, and neuroinflammation (Twarowski and Herbet, 2023) in the central nervous system (CNS). The innate immune system cells are vital in regulating brain homeostasis and can eliminate Aβ plaques and aggregated Tau proteins, while abnormal activation of these cells can result in programmed cell death, release of proinflammatory cytokines and consequently chronic neuroinflammation, as well as inhibit phagocytosis of Aβ aggregates and lead to Aβ aggregates accumulation. The inflammatory responses are mainly carried out by two key glial cells, including microglia and astrocytes (Rajesh and Kanneganti, 2022). The abnormal activation of microglia and astrocyte can release cytokines which can activate downstream target genes like NF-κB and AP-1 and elicit neurotoxicity like neuronal death (Singh, 2022). It is a complex condition that involves many pathological pathways from synaptic loss, deficient neurotransmission, progressive accumulation of amyloid proteins to tau toxicity (Khan et al., 2020). However, there is still no cure for AD. FDA has aproved 5 drugs for AD, including donepezil (Adlimoghaddam et al., 2018), rivastigmine, galantamine, memantine, and combine donepezil with memantine (Joe and Ringman, 2019). But they can only relieve symptoms without any disease-modifying effects. It is under great demand to find efficacious strategies that can significantly prevent or slow the progression of AD (Passeri et al., 2022). At the moment, a number of promising therapeutic strategies are undergoing clinical trials, including synaptic dysfunction modifying agents, neurotransmission modifying agents, anti-amyloid agents, anti-tau agents (Cummings et al., 2019), as well as anti-inflammatory agents. Due to the complicated nature of AD, it is more promising to seek for multitarget therapies which can be more beneficial by interfering with multiple pathological processes (Cummings et al., 2022; Ju and Tam, 2022). Herbal medicine is one of these therapies for AD. AD can be attributed to a combination of multiple factors, including unmodifiable risk factors, such as age, genetics, and family history of AD, as well as modifiable risk factors, such as physical activity (Sabia et al., 2017), smoking, low education level, body weight, and blood sugar (Livingston et al., 2020). Gut microbiota dysbiosis has attracted much attention as a novel therapeutic target for AD in the last few years. In this review, we will focus on the possible connections between AD, microbiota dysbiosis and herbal medicine.

## 2 Relationship between the gut microbiota and brain functions: the microbiota-gut-brain axis

**FIGURE 1:**
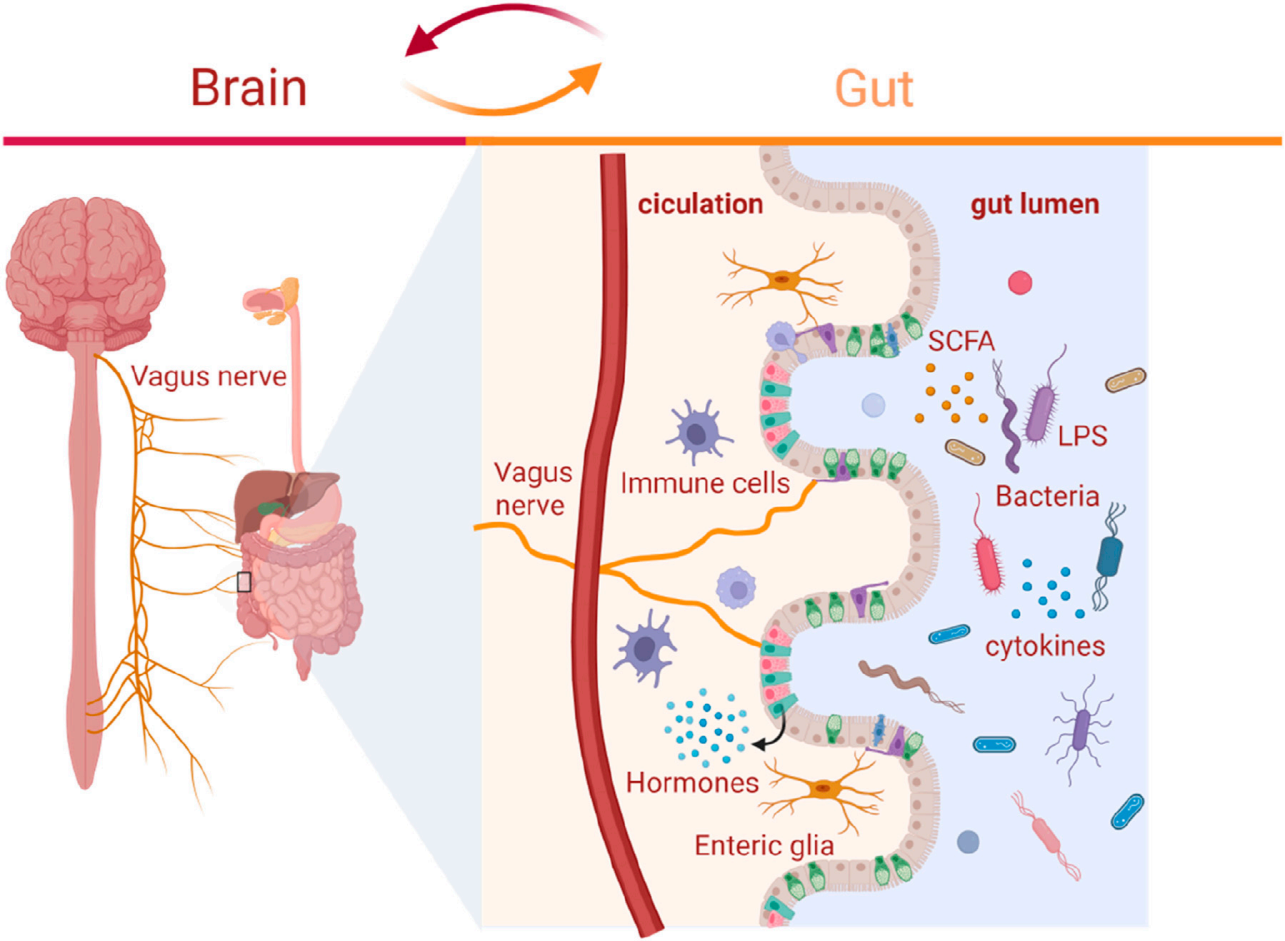
The microbiota-gut-brain axis. The microbiota-gut-brain axis is a bidirectional communication system involving neural, immune, endocrine and metabolic pathways. 1) Immune pathways: The lipids, LPS and lipoproteins on the bacterial wall act as specific antigens called PAMPs which activate PRRs, such as TLRs, and lead to activation of immune cells and cytokine production; 2) Endocrine pathways: The enteroendocrine cells in the gut epithelium secrete hormones when stimulated by various substances in the lumen; 3) Neural pathways: The gut sends signals from the periphery via the ENS which contains sensory and motor neurons to the brain, and the brain sends instructions back to the effector cells in the gut. The vagus nerve is also involved in the neural transmission between the brain and the ENS. Metabolites or products from gut bacteria stimulate the vagus nerve through the ENS and further influence the brain; 4) Metabolic pathways: The gut microbiota produce substances, such as SCFAs, which can influence brain functions. LPS: lipopolysaccharides; SCFAs: short-chain fatty acids.

The interaction between the gut microbiome, the gut and the brain are bidirectional and called the microbiota-gut-brain axis (Mayer et al., 2022). The microbiota-gut-brain axis encompass immune, endocrine, neural, and metabolic pathways (Novotný et al., 2019). 1) Immune pathways: Lipids, lipopolysaccharides (LPS) and lipoproteins on the bacterial wall act as specific antigens called pathogen-associated molecular pattern molecules (PAMPs), which activate pattern recognition receptors (PRRs), such as Toll-like receptors (TLRs), and lead to activation of immune response and cytokine production (Mcclure and Massari, 2014); 2) Endocrine pathways: The enteroendocrine cells in the gut epithelium secrete hormones when stimulated by various substances in the lumen, such as serotonin, histamine and dopamine which will bind to receptors expressed by various tissues; 3) Neural pathways: The gut sends signals from the periphery via the enteric nervous system (ENS) which contains sensory and motor neurons to the brain, and the brain sends instructions back to the effector cells in the gut. The vagus nerve is also involved in the neural transmission between the brain and the ENS. Metabolites or products from gut bacteria stimulate the vagus nerve through the ENS and further influence the brain; 4) Metabolic pathways: The gut microbiota produces substances, such as short-chain fatty acids (SCFAs), which can influence the brain functions (Martin-Gallausiaux et al., 2021). Signals from the gut, mainly including immune signaling molecules, host-derived signaling molecules, and diet-derived molecules can be transported to the brain via the systemic circulation directly or indirectly through interacting with intestinal cells and microbiota in the gut. Intestinal cells and gut microbiota can produce and release molecules locally acting on vagal afferents and modulating sympathetic and parasympathetic activities as well as the hypothalamic-pituitary-adrenal (HPA) axis. The roles of this complicated system mainly involve monitoring and integrating intestinal functions as well as connecting with the cognitive centers of the brain through immune activation, enteric reflex, entero-endocrine pathways, and regulating intestinal permeability (Margolis et al., 2021). The structure of microbiota-gut-brain axis is shown in Figure 1.

A collection of approximately 100 trillion microbes dwell within the gut of the host, and most of them belong to bacteria, others include viruses, fungi, and protozoa. These microbes with their metabolites together play essential roles in regulating host health and development of various diseases by participating in various physiological and pathological processes (Adak and Khan, 2019; Ling et al., 2022). The majority of gut microbiota belongs to two phyla, the *Bacteroidetes* and the *Firmicutes*. The rest of them belong to the *Proteobacteria*, *Actinobacteria*, *Fusobacteria* and *Verrucomicrobia*, which can be categorized into beneficial, neutral, and detrimental microbiota in general based on their impacts. The metabolites and products from microbiota are also divided into beneficial and harmful ones. For example, SCFAs produced by specific phyla, such as butyrate, propionate, and acetate, are imperative due to their anti-inflammatory activities (Ho et al., 2017). SCFAs promote the production of tight junction proteins that are important for maintaining a tight and selective barrier function. Evidence has shown that a higher abundance of butyrate-producing bacteria and higher butyrate concentrations could be protective against enteric infection in rodents (Osbelt et al., 2020). While microbiota-derived amyloids can exert prion-like properties and other pathogenic microbial metabolites produced by the gut microbiota, such as LPS, are intensely associated with neuroinflammation and AD neuropathologies (Zhao et al., 2017). In a healthy gut, the abundance, diversity and composition of microbiota is relatively stable, which brings no harm to the host. Under normal circumstances, the state of well-balanced microbiota plays a vital role in maintaining the permeability of the intestinal membrane by regulating the epithelial barrier, which blocks the transgression of pro-inflammatory cytokines produced in the gut into the circulation system.

## 3 Gut microbiota dysbiosis and Alzheimer’s disease

Gut microbiota dysbiosis refers to quantitative and qualitative changes of microbiota in the gut, like the reduction in beneficial microbiota, such as anti-inflammatory bacteria, and increase in detrimental microbiota, such as pro-inflammatory bacteria (Yang et al., 2020).

Changes in the composition of the gut microbiota in AD patients have been observed in different studies and their results varied from distinctly enriched Enterobacteriacea, *Bacteroides*, Actinobacteria, Ruminococcus, Lachnospiraceae, and Selenomonadales to decrease of *Bacteroides*, and increase of Prevotella. While other analysis revealed that AD patients showed a decreased abundance of Firmicutes and Bifidobacterium, along with increased abundance of Bacteroidetes (Vogt et al., 2017). Gut dysbiosis exacerbated AD pathologies and cognitive deficits by activating the Ccaat/Enhancer binding proteinβ(C/EBPβ)/AEP signaling pathway in mouse models (Chen et al., 2022), which was documented to mediate neuroinflammation in AD pathologies (Wang et al., 2018). Data from Aβ precursor protein (APP) transgenic mouse model supported the essential role of gut microbiota in AD development as a remarkable shift in the gut microbiota was accompanied with an obvious reduction in cerebral Aβ amyloid pathology (Harach et al., 2017). In conditions of dysbiosis, compounds produced by gut bacteria activated TREMs on macrophages, resulting in pro-inflammatory responses which not only damaged the gut barrier, but also promoted a systemic inflammatory response via the microbiota-gut-brain axis, leading to impairment of the blood-brain barrier and neuroinflammation (Natale et al., 2019). Gut dysbiosis is directly and closely related to a “leaky gut,” manifested by gut barrier dysfunction and impaired gut permeability, which subsequently leads to an increased risk of AD (Spielman et al., 2018). In a state of dysbiosis, the level of benificial products like SCFAs decreases while deleterious substances like LPS increases, the integrity of the epithelial barrier is broken, leading to increased intestinal permeability and subsequent systemic inflammation (Al et al., 2020; El-Sayed et al., 2021). Ultimately, this results in neuroinflammation and the formation of amyloid plaques. In the meantime, the microbial-derived amyloids can translocate through the impaired epithelial barrier, and cross-seed with endogenous amyloids, leading to aggravation of neuroinflammation, amyloid deposition, amyloidosis and AD progression (Leblhuber et al., 2020; Bairamian et al., 2022).

Chronic neuroinflammation, along with systemic inflammation is closely associated with amyloidosis and AD progression (Mou et al., 2022). Growing evidence shows that gut microbiota dysbiosis has important influence on AD pathogenesis (Leblhuber et al., 2020) by increasing the secretion of LPS and amyloids (Lin et al., 2019), permeability of the intestinal membrane and the blood-brain barrier, promoting oxidative stress, neuroinflammation, Aβ formation, and ultimately neuronal death (Bonfili et al., 2021; Janeiro et al., 2022). Overall, gut dysbiosis can influence both central and peripheral pathological processes, thereafter contributing to the pathogenesis and progression of AD (Ma et al., 2019; Liu et al., 2020; Sun et al., 2020; Weber et al., 2023). Therefore, maintaining a balanced or healthy state of gut microbiota composition is crucial for ameliorating peripheral low-grade inflammation, suppressing neuroinflammatory stimuli and exerting beneficial effects on AD (Bonfili et al., 2021; El-Sayed et al., 2021; Shabbir et al., 2021).

**FIGURE 2:**
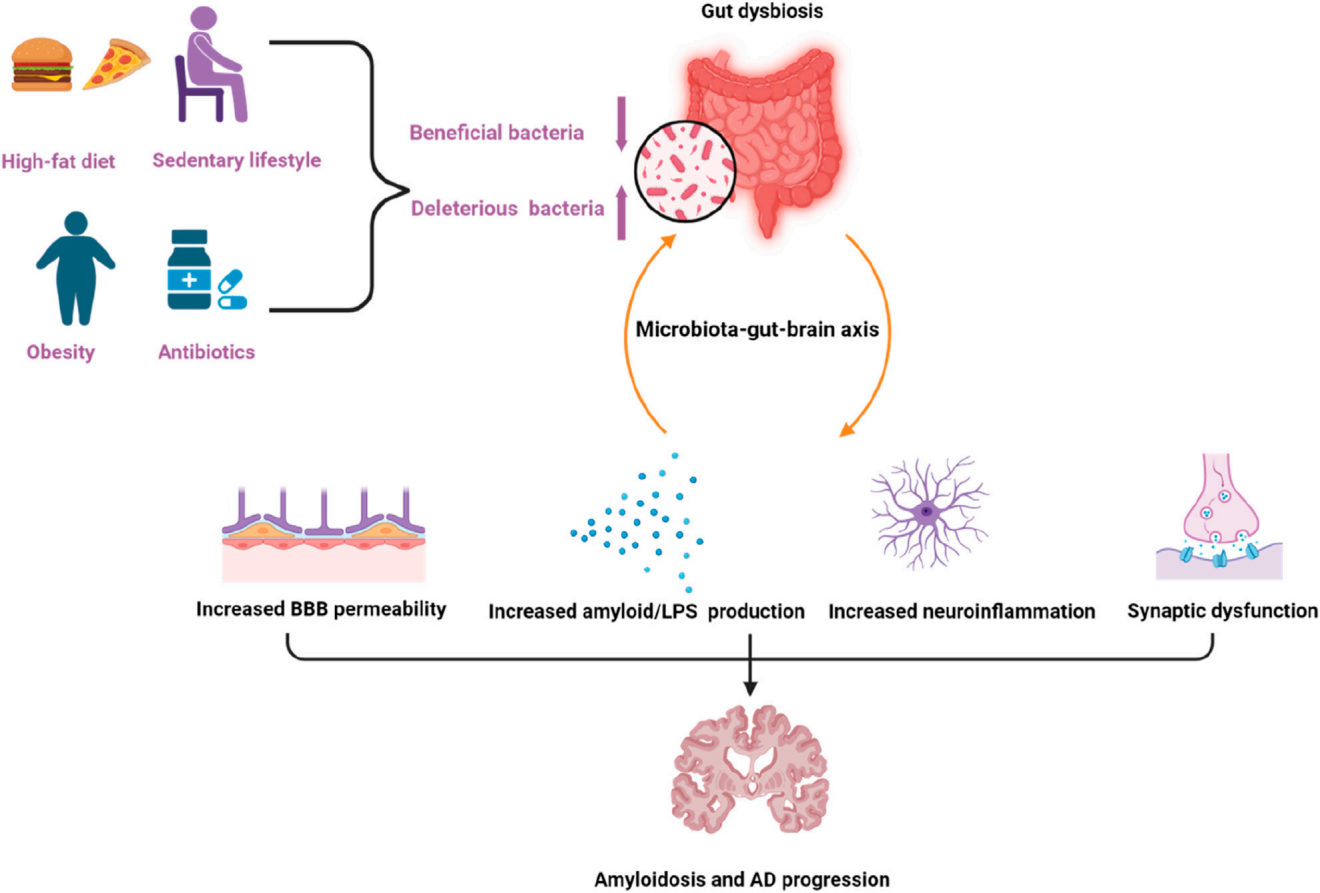
Factors that can influence gut dysbiosis and the relationship between gut microbiota dysbiosis and Alzheimer’s disease. Factors like a high-fat diet, obesity, a sedentary lifestyle, and antibiotics can contribute to gut dysbiosis. Dysbiosis of gut microbiota contributes to amyloidosis and AD pathologies through the microbiota–gut–brain axis via multiple processes, including increasing the permeability of the blood brain barrier (BBB), amyloid/LPS production, neuroinflammation as well as synaptic dysfunction. The purple arrow pointing upwards indicates upregulation, the other one indicates downregulation.

The composition of gut microbiota undergoes dynamic changes due to endogenous or exogenous factors, such as dietary pattern, environmental factors, lifestyle, diseases, and medications (El-Sayed et al., 2021). An increased intake of a high-energy or low-fiber diet, obesity (Grigor Eva, 2021), a sedentary lifestyle (O'Toole and Shiels, 2020), and antibiotic treatment can contribute to gut dysbiosis (Lee et al., 2022). Current evidence has shown that diet and dietary components can exert modulatory effects on the composition of the gut microbiota. Specific dietary components like fat or sugar abundant in the Western diet affect microbial composition in a negative manner and are related to chronic low-grade inflammation, while other dietary components like fiber or resistant starch rich in plant-based and Mediterranean diet are capable of shifting the microbial composition in a positive manner (Beam et al., 2021). Factors that influence gut dysbiosis and the relationship between gut microbiota dysbiosis and AD were shown in Figure 2.

## 4 Herbal medicine and AD

Given the intense interaction between gut microbiota and AD, more attention has been paid to searching for strategies targeting the gut microbiota to prevent or delay the onset and progression of AD. A number of potent therapeutic approaches have been elucidated to target the gut microbiome, such as prebiotics, probiotics, dietary agents, and herbal medicine (Peterson, 2020). According to the definiton by World Health Organization (WHO), herbal medicine covers complete herbs, herbal materials, herbal formulations, and end herbal products containing bioactive compounds derived from plants. A large number of studies have already shown that herbal medicine possesses multitarget therapeutic effects (Cui et al., 2017; Sahoo et al., 2018; Guo et al., 2019; Raghuvanshi et al., 2021; Fernandes et al., 2022), which can relieve the symptoms of AD. The neuroprotective potential of seven traditional medicinal plants were evaluated in two different human brain cell lines and the results showed that they were able to scavenge free radicals and inhibit the glycogen synthase kinase-3β (Fernandes et al., 2022). Aβ_25-35_ and D-gal-induced AD rats and Aβ_25-35_-induced PC12 cells were applied to investigated the anti-AD effects of Kai-Xin-San, and the results demonstrated that KXS could be effective in ameliorating AD through multiple mechanisms including regulating neurotransmitters and the PI3K/Akt signal pathway (Guo et al., 2019). An array of plant extracts and their secondary metabolites were tested *in vitro* for their inhibitory roles on acetylcholinesterase (AChE), butyrylcholinesterase (BChE), and beta-site amyloid precursor protein cleaving enzyme 1 (BACE-1), and the most active one was further studied *in vivo* in mouse models. The results supported that Woodfordia fruticosa (L.) Kurz can be a lead anti-AD candidate and indicated its potential as a plant-based drug for AD (Raghuvanshi et al., 2021). Matrine could inhibit Aβ42-induced cytotoxicity and suppress the Aβ/RAGE signaling pathway *in vitro* and decrease the level of proinflammatory cytokines and Aβ deposition in AD transgenic mice, suggesting that it might be a novel anti-AD compound with multi-target effects (Cui et al., 2017).

**FIGURE 3:**
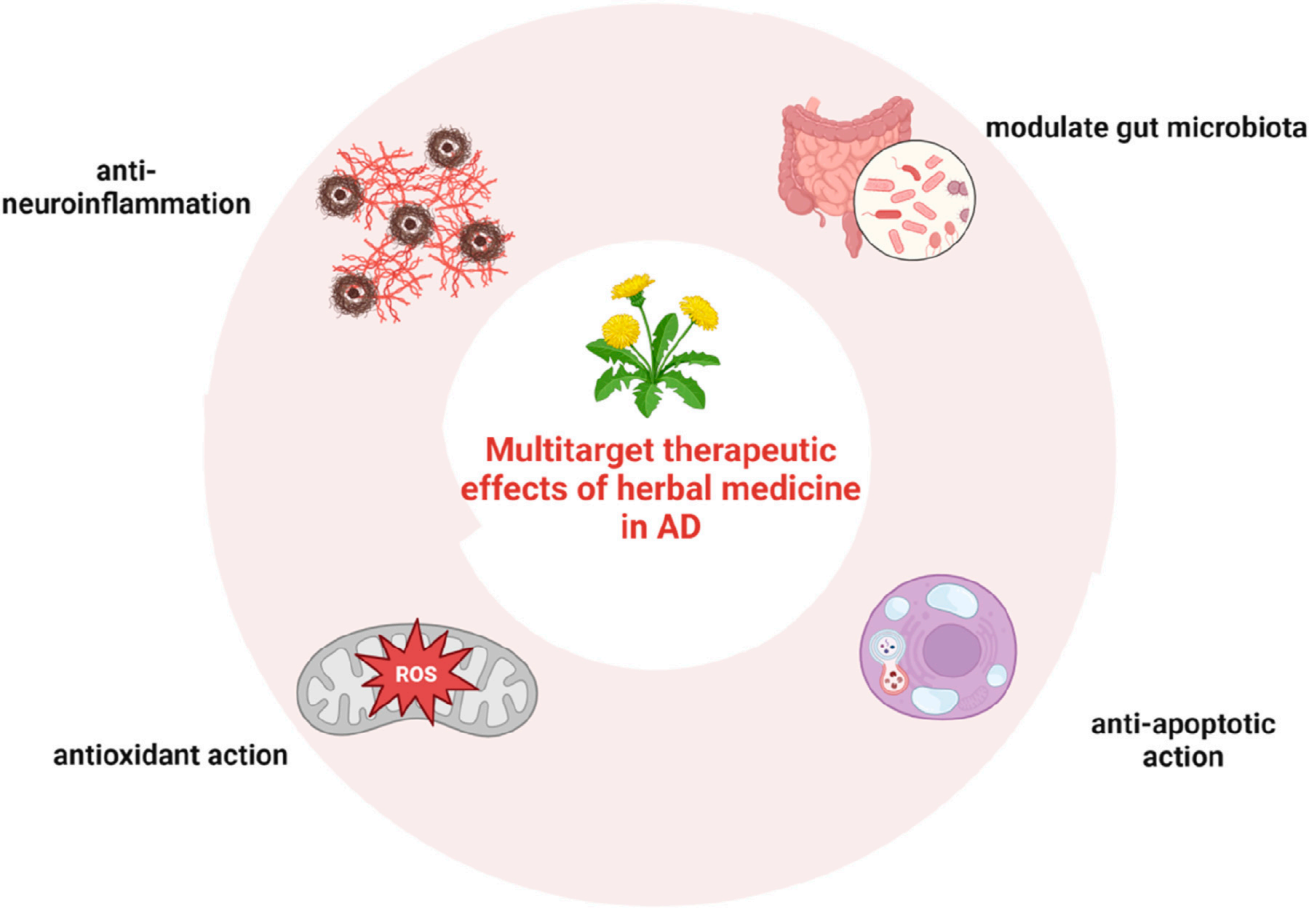
Multitarget therapeutic effects of herbal medicine in AD, including anti-inflammatory, anti-apoptotic, and antioxidant actions, as well as the regulatory effect on the composition of gut microbiota.

Herbal formula is the basic prescription made up of a mixture of some specific herbal medicine plants, which contains substantial chemical constituents. The complex composition of compounds in herbal formulae may also provide a basis for the multi-target interactions between herbs and AD. 125 anti-AD traditional Chinese medicine formulae and their chemical compounds were collected from databases and the results of computational prediction, molecular docking and biological validation showed that 12 compounds including Nonivamide, Bavachromene and 3,4-Dimethoxycinnamic acid possessed multi-target anti-AD activities (Zhang B. et al., 2021). The possible mechanisms for their effects involve anti-inflammatory, anti-apoptotic, and antioxidant actions, through which neurons are protected from amyloid-β damage, amyloid-β secretion inhibited, and subsequent oxidative stress and apoptosis suppressed (John et al., 2022). In this regard, herbal medicine may have the potential to overcome the limitations of conventional medicines. In recent years, it has been found that herbal medicine is also capable of regulating the composition of gut microbiota (Dingeo et al., 2020; Gong et al., 2020; Zhao et al., 2021). The multitarget therapeutic effects of herbal medicine in AD are shown in Figure 3.

Databases including PubMed, Wiley Online Library, ScienceDirect, Web of Science, Scopus, Cochrane Review, and China National Knowledge Infrastructure, were used in our review to obtain relevant studies, and we searched for literatures using key words like “herb” or “herbal medicine “or” herbal formula” or “plant,” and “microbiota,” and “AD” or “Alzheimer’s Disease” and refined the publication date to the past 10 years.

Here, firstly, we discussed commomly used herbs or herbal preparations for AD and their potential neuroprotective mechanisms. Meanwhile, evidence showed that some preparations also had intense connections with gut microbiota. It is reasonable to suspect that the effect on gut microbiota may be an essential part of neuroprotective mechanisms against AD.

### 4.1 Commomly used herbs or herbal preparations for treatment of AD

#### 4.1.1 Yang Xin Tang

Yang Xin Tang (YXT), a traditional Chinese herbal preparation recorded in Gujin Yitong Daquan, exerts protective effects for AD patients and the underlying mechanisms are elucidated in an *in vitro* study, including inhibiting β-amyloid aggregation, β-amyloid-induced cytotoxicity and activities of the acetylcholinesterase (Lo et al., 2023). YXT and its herbal components can be a potential medicine for treating AD as they are able to exert multi-target regulatory effects in AD pathogenesis. The involvement of gut microbiota in the multi-target effects of YXT on AD has not been elucidated yet in existing studies.

#### 4.1.2 Yi-Gan-San

Yi-Gan-San, a traditional Chinese prescription from Baoying Cuoyao used for the treatment of AD, possesses neuroprotective effects, which contain plenty of chemical compounds, mainly triterpenes, flavonoids, phenolics, and alkaloids (Ishida et al., 2022). Mechanisms underlying its neuroprotective actions in AD is not fully comprehended. Results of an *in vivo* study on Yi-Gan-San in hTauR406W transgenic flies, a *Drosophila* AD model with Tau-induced toxicity, showed that Yi-Gan-San can ameliorate AD by decreasing p-Tau expression and alleviating tauopathy (Su et al., 2022). YGS40, a fraction of Yi-Gan San, could alleviate hydrogen peroxide-induced apoptosis in PC12 cells and may be beneficial for decreasing oxidative stress-induced apoptosis in AD (Zhao et al., 2015). YGS could also suppress Aβ oligomer-induced neuronal apoptosis in primary cultured cortical neurons (Kanno et al., 2013), and the effect was partly attributed to suppressing the apoptosis effector caspase-3 (Kanno et al., 2015). Up to now, all of the known chemical constituents isolated from YGS and their chemical structures were proposed in an review (Yang et al., 2023). In the field of clinical research, YGS is usually used in the treatment of AD, however, further comprehensive studies on its chemical constituents and quality assessments are needed to evaluate the efficacy of this herbal formula in treating AD patients. There is no evidence demonstrating the effect of YGS on the gut microbiota yet, however, the pharmacokinetics of the components of YGS could be affected by gut microbiota (Ishida et al., 2022).

#### 4.1.3 Jiedu-Yizhi formula

Jiedu-Yizhi formula (JDYZF), a classical formula deccribed in Zhong Yi Fang Ji Da Ci Dian, has been used for AD patients in the clinic for years, while the detailed mechanism underlying its therapeutic effect still needs to be illustrated. In an Aβ _25-35_-induced rat model, JDYZF showed multiple beneficial effects by attenuating neuronal loss in the hippocampus, decreasing the deposition of Aβ, inhibiting the expression of proteins associated with pyroptosis, and reducing the levels of IL-1β and IL-18 (Wang et al., 2022a), which provided scientific evidence for using JDYZF to treat AD patients.

Meanwhile, JDYZF increased the diversity of the gut microbiota in AD rats including increasing the abundances of *Lactobacillus*, Prevotella, decreasing the abundance of Turicibacter, Desulfovibrio, *Helicobacter* (Wang et al., 2022b). JDYZF increased the diversity of the gut microbiota in AD rats by affecting the abundances of different strains of bacteria at the phylum level, genus level and species level, leading to alleviation of neuroinflammation in AD rats. Another study on the APP/PS1 mouse model showed that JDYZF treatment could also increase the enrichment of *Lachnospiraceae, Ruminococcaceae*, and *Actinobacteria*, and decrease the enrichment of *Alistipes* and *Muribaculaceae* (Yang et al., 2021). The improved cognition might be attributed to its modulating effect on the gut microbiota.

#### 4.1.4 Shenqi Yizhi granule

Shenqi Yizhi granule (SQYG) is a Chinese herbal formula that has been proven to be effective and applied to treat AD in clinical trials and animal models. SQYG treatment exhibited significant neuroprotective effects on the hippocampus of 5XFAD mice through modulating multiple pathological processes, such as synaptic transmission, signal transduction, and amino acid metabolism (Ren et al., 2020). Other relevant mechanisms involve inhibiting response of astrocytes regulated by the JAK2/STAT3 signaling pathway, which has been proven in an Aβ_1-42_ -induced rat model of AD (Li P. et al., 2021). Other possible molecular mechanisms of SQYG were investigated through an integration strategy of network pharmacology and molecular docking. The results showed that therapeutic mechanisms of SQYG involved regulating lipid metabolism, metal ion metabolism, interleukin signaling pathways, and neurotransmitter receptor mediated signaling pathways (An et al., 2018; Wang L. et al., 2022). Whether SQYG has a modulatory effects on gut microbiota and their metabolites is one of the specific mechanisms influencing AD progression that still need to be further investigated.

#### 4.1.5 Cordyceps militaris extracts and cordycepin

Cordycepin is the major bioactive component extracted from Cordyceps militaris (CM) that is a well-known traditional Chinese medicine. A previous comparative study demonstrated that cordycepin significantly increased the expression level of 5-hydroxymethylcytosine and downregulated the transcription level of Apolipoprotein E (ApoE), thereby hampering the formation of neurofibrillary tangles and the accumulation of Aβ (Cao D. et al., 2020). These results indicated that cordycepin may delay the progression of AD. Analysis on gut microbiome and metabolomics showed that CM extracts and cordycepin could downregulate oxidative stress biomarkers and inflammatory cytokines by modulating gut microbiota to the state with a high abundance of *Firmicutes/Bacteroidetes* (Liu et al., 2023). In a pig model, CM could modulate the intestinal barrier function by improving intestinal morphology, elevating the expression of zona occluden-1, claudin-1, down-regulating pro-inflammatory cytokines and up-regulating anti-inflammatory cytokines due to its effect on suppressing the expression of key proteins of the TLR4/NF-κB signaling pathway. It also changed the gut microbial composition and increased the levels of acetate and butyrate (Zhang P. et al., 2023). The modulatory effects of cordycepin on gut microbiota and metabolites may be one of the specific mechanisms influencing AD progression, which still needs to be further confirmed.

#### 4.1.6 Bushen-Huatan-Yizhi formula

Bushen-Huatan-Yizhi formula (BSHTYZ) is used to treat AD patients in the clinic. Animal studies revealed that BSHTYZ improved spatial learning and memory by affecting specific molecular targets involved in AD, such as the glycogen synthase kinase-3β(GSK-3β)/cAMP response element binding protein (CREB) signaling pathway (Yang et al., 2021). Another study showed that Bushen Huatan formula can increase the level of short chain fatty acid and improve the function of the intestinal barrier by regulating gut microbiota, increasing the abundance of *Proteus* and *actinomycetes, Clostridium, Lactococcus* (Cui et al., 2023). The modulatory effect on gut microbiota by BSHTYZ may be one of the possible neuroprotective mechanisms in delaying the progression of AD, which still needs to be further investigated.

#### 4.1.7 Huanglian Jiedu decoction

Huanglian Jiedu decoction (HLJDD), a heat-clearing formula from Waitai Miyao Fang, is also commonly used to treat AD. A previous study investigated the protective mechanism of this formula in managing AD model rats by analysing the pharmacokinetics of bioactive ingredients and showed that it could exert neuroprotective effects through regulating the levels of inflammatory cytokines and thus alleviating the central inflammatory status of AD (Gu et al., 2018).HLJDD could also mitigate abnormal sphingolipid metabolismis which is intensely involved in the occurrence and development of AD (Qi et al., 2022). HLJDD altered the gut microbiota in Tg-APP/PS1 mice by decreasing the abundance of *Firmicutes* and increasing the abundance of *Proteobacteria* at the phylum level, increasing the relative abundances of *Prevotellaceae, Lactobacillaceae, Peptococcaceae*, reducing the relative abundance of *Bacteroidales_S24-7_group, Lachnospiraceae* and *Porphyromonadaceae* at the family level (Gu et al., 2021). A recent study further elucidated the interaction between bioactive ingredients in HLJDD and AD-related targets through comprehensive network pharmacology and molecular docking analysis. The results provided the evidence that HLJDD can effectively treat AD through multiple targets and multiple pathways including regulating gut microbiota homeostasis (Zheng et al., 2023).

#### 4.1.8 SuanZaoRen decoction

SuanZaoRen Decoction (SZRD), a tranquillizing formula from Jinkui Yaolue, is widely used to treat AD. Its anti-AD molecular mechanisms involve alleviating Aβ deposition, neuronal loss, synaptic loss and iron accumulation by regulating the DJ-1/Nrf2 signaling pathway in AD mice (Long et al., 2024). Other evidence showed that SZRD could regulate microbiota-gut-brain axis by inhibiting the TLR4/NFκB/NLRP3 pathway so that dysbiosis of the gut microbiota in the depressive mouse model was attenuated, restoring the relative abundance of gut microbiota by decreasing the abundance of *Bacteroidetes* and increasing the *Firmicutes* phylum members (Du et al., 2023). Based on results from bioinformatic analysis, network pharmacology, molecular docking, and molecular dynamics simulation, SZRD may improve cognitive performance of AD patients with diabetes through a number of potential bioactive ingredients including licochalcone A, isorhamnetin, kaempferol, quercetin, and formononetin (Chen et al., 2024).

#### 4.1.9 *Centella asiatica* (CA)

ECa 233 is a standardized extract from CA and evidence has shown that ECa 233 exerts a therapeutic effect on the fear memory deficit and synaptic dysfunction in AD model mice (Pairojana et al., 2023). This might be attributed to its anti-inflammatory effect as shown in an *in vitro* study (Sukketsiri et al., 2019), as well as the possible anti-inflammatory mechanism through which impaired intestinal mucosal barrier is restored through modulating gut microbiota composition. The latter could be due to the decreased abundance of harmful genera, such as *Staphylococcus* (Li H. et al., 2021). More research is needed to further verify the importance of CA and its extract on gut microbiota dysbiosis and the subsequent influence on AD.

#### 4.1.10 Naoxintong capsule

Naoxintong (NXT) capsule is a Chinese medicine already used in the clinic for AD patients, which is a powder mixture of multiple herbs without any extraction. NXT treatment in APP/PS1 mice showed neuroprotective effects and the potential mechanisms included regulating inflammatory cytokines and consequently decreasing p-Tau and Aβ accumulation, attenuating neuronal apoptosis in the brain (Wang X. et al., 2021). Previous studies have shown that Naoxintong capsules are able to increase the diversity of gut microbiota, to influence the composition of microbiota, as well as to reverse the increase in the ratio of the Firmicutes to Bacteroidetes in relative abundance in animals (Zhang et al., 2019; Lu Y. et al., 2022). This may partly provide an explanation for the potential protective effects of NXT on AD from another perspective.

#### 4.1.11 Kai-Xin-San

Kai-Xin-San (KXS) is a Traditional Chinese Medicine formula that has been applied to treat AD patients for over 100 years, which originally was recorded in Beiji Qianjin Yao Fang written by Sun Simiao. The efficacy and potential pharmacological mechanisms of KXS against AD were evaluated and proven to involve multiple targets, multi components and multiple pathways (Luo et al., 2020; Yi et al., 2020). KXS improved cognitive function via regulating SIRT3-mediated neuronal cell apoptosis (Su et al., 2023), activating the Wnt/beta-catenin signaling pathway in APP/PS1 mice and AD rats (Shan et al., 2023; Xu et al., 2023). Network pharmacology analysis validated the multi-component and multi-target therapeutic mechanisms of KXS for treatment of AD, involving inhibiting Tau protein hyperphosphorylation, inflammation, and apoptosis (Jiao et al., 2022; Wang et al., 2023). KXS also changed the composition of gut microbiota, significantly reversing the decreased abundance of *Bifidobacterium* and *Helicobacter* in the gut, which is accompanied by antidepressant-like effects in stressed animal models (Cao C. et al., 2020). It is possible that modulation of the composition of gut microbiota may also be one of the essential aspects of KXS against AD, exerting positive effects through regulating the gut-brain axis.

#### 4.1.12 Cu-zhi-2-hao-fang (CZ2HF)

Cu-zhi-2-hao-fang (CZ2HF) is a traditional Chinese medicine used clinically for the treatment of amnesia. It improved cognitive impairment and neuronal injury in rats by reducing the expression of TNF-α, IL-1β, COX-2, decreasing the Bax/Bcl-2 ratio, levels of Aβ1-42 and active-caspase-3, as well as IκB-αdegradation and p-NF-κB p65 activation (Zeng et al., 2019). The pharmacological effects of CZ2HF decoction on AD were elucidated by network analysis, 914 compounds were identified and 9 of them were mainly involved in the pathologic process of AD including itosterol, beta‐sitosterol, stigmasterol, and luteolin. The key genes they target include TNF, ICAM1, MMP9 and IL‐10. This provided further insight into the mechanisms of CZ2HF managing AD (Wei et al., 2021). However, there is little evidence on the effect of CZ2HF on gut microbiota, which may be worthy of further investigation.

#### 4.1.13 Morinda Officinalis

Bajijiasu (BJJS) is extracted from Morinda Officinalis, a Chinese herb. It exerted neuroprotective effects against cognitive impairment in APP/PS1 double transgenic mice by regulating the metabolism of amyloid-β, enhancing the expression of neurotrophic factors, inhibiting neuroinflammation and elevating the expression of synapse structure related proteins (Cai et al., 2017). Oligosaccharides from Morinda officinalis (OMO) are effective in alleviating AD by improving gut microbiota based on their capacity in increasing the abundance of *Lactobacillus, Allobaculum, Lactobacillaceae,* and *Lachnospiraceae* in APP/PS1 transgenic mice, indicating their prebiotic role in AD animal models (Xin et al., 2018).

#### 4.1.14 Huperzine A

Huperzine A (HupA), an alkaloid derived from club moss Huperzia serrata, has been used for treating AD for centuries in Chinese medicine due to its effect on inhibiting acetylcholinesterase. The integrated overview of the molecular mechanisms of HupA in AD showed the enhanced Wnt signaling, downregulation of the activity of GSK-3β, upregulation of Bcl-2 and neurotrophin, and downregulation of Bax (Friedli and Inestrosa, 2021). However, there is little evidence on the effects of HupA on gut microbiota in AD models.

#### 4.1.15 GuanXinNing tablet

GuanXinNing Tablet (GXN) is an oral preparation composed of two Chinese herbs, Ligusticum chuanxiong Hort and Salvia miltiorrhiza Bunge. GXN improved memory and behaviors in AD rabbits through enhancing neuronal metabolism, inhibiting oxidative stress and neuronal apoptosis, as well as ameliorating the changes in the gut microbiota by decreasing the *Firmicutes/Bacteroidetes* ratio and improving the abundances of *Akkermansia and dgA-11_gut_group* (Zhang F. et al., 2021)*.* Ligusticum chuanxiong Hort, also called Chuanxiong (CX) in China, is a long-standing medicinal herb widely used in China. One of active components of CX is Tetramethylpyrazine (TMP), a major alkaloid which is evidenced to exert neuroprotective effects on AD (Meng et al., 2021). This is achiveved by reducing the accumulation of tau and amyloid-βin rats (Zhang Q. et al., 2021), inhibiting GSK-3β (Lu et al., 2017), halting the inflammatory progression through inhibiting the production of inflammatory cytokines in primary microglial cells (Kim et al., 2014). Salvia miltiorrhiza Bunge, also called Danshen (DS) in China, is also a famous Chinese medicine. DS could improve chronic sleep deprivation-induced cognitive dysfunction in rats through regulating the composition of the gut microbiota, preserving functions of the gut and the blood brain barriers, and suppressing inflammation mediated by the LPS-TLR4 signaling pathway. DS decreased the number of harmful bacteria, such as *Corynebacterium, Blautia*, *Rhodococcus*, *Allobaculum* and increased the number of beneficial bacteria, such as *Alloprevotella*, *Bacteroides*, *Lactobacillus* (Yin et al., 2024).

#### 4.1.16 Gastrodia elata Blume

Data from previous studies suggested Gastrodia elata Blume (tianma) may be used as a potential complementary therapy for AD (Heese, 2020). P-hydroxybenzaldehyde (p-HBA), one of the major active components of Gastrodia elata, had a therapeutic effect on AD by alleviating Aβ-induced toxicity via enhancing antioxidant and anti-aging activities and inhibiting Aβ aggregation, as well as enhancing anti-oxidative stress (Yu et al., 2024). A bioactivity test showed that a polysaccharide from Gastrodia elata, named GEP-1, could modulate gut microbiota by promoting the growth of *A. muciniphila (A. muciniphila)* and *Lacticaseibacillus paracasei (L.paracasei)* strains (Huo et al., 2021). In addition, another active ingredient from G. elata, parishin, could prevent the loss of tight junction proteins, modulate the composition of gut microbiota to a healthier state in aged mice (Gong et al., 2023). It could alter microbial diversity and decrease the aberrant abundance of pathogenic bacteria, such as *Turicibacter* and *Erysipelatoclostridium* (Zhao et al., 2022).

Gastrodin (Gas), also an active ingredient from G. elata, is known to exhibit neuroprotective effects on AD. Gas reversed the memory dysfunction in D-gal-administered AD mouse model via partly modulating the microbiota-gut-brain axis with a positive link with *Firmicutes* and had a negative link with *Cyanobacteria, Proteobacteria,* and *Deferribaceters* (Fasina et al., 2022). Gas treatment improved cognitive deficits and Aβ deposition in APP/PS1 mice by inhibiting neuronal apoptosis via increasing the level of Bcl-2 and decreasing the level of Bax, increasing the expression levels of insulin-like growth factor-1(IGF-1) and cAMP response element-binding protein (CREB) in APP/PS1 mice, as well as rectifying the abnormal composition and structure of gut microbiota (Zhang Y. et al., 2023). These studies provide scientific basis for neuroprotective roles of G. elata in AD and demonstrate the relevant mechanisms which include modulation of the gut microbiota. Examples of herbs or herbal preparations for treatment of AD, their targeted and molecular basis, biological pathways, and the connections with gut microbiota are shown in Table 1.

**TABLE 1:** Examples of herbs or herbal preparations for treatment of AD, their targeted and molecular basis, biological pathways, and the connections with gut microbiota.

Herbs or preparations	Targeted and molecular basis, biological pathways in AD	Effects on gut microbiota	Models	References
Yang Xin Tang	Inhibit β-amyloid aggregation, β-amyloid-induced cytotoxicity and acetylcholine esterase	Not studied yet	*In vitro*	Lo et al. (2023)
Yi-Gan-San	(1) Alleviate tauopathy (2) Alleviate hydrogen peroxide-induced apoptosis (3) suppress Aβ oligomer-induced neuronal apoptosis (4) suppress the apoptosis effector caspase-3	Not studied yet	Transgenic flies; *in vitro* cell lines	Kanno et al. (2013), Kanno et al. (2015), Zhao et al. (2015), Ishida et al. (2022), Su et al. (2022), Yang et al. (2023)
Jiedu-Yizhi formula	(1) Attenuate neuronal loss in the hippocampus (2) decrease the deposition of Aβ (3) inhibit the expression of proteins associated with the pyroptosis pathway and reducing levels of IL-1β and IL-18	(1) Increase the richness of Lachnospiraceae, Ruminococcaceae, and Actinobacteria and decrease the richness of Alistipes and Muribaculaceae (2) Affect the abundances of different strains of bacteria at the phylum level, genus level and species level	Rats/mice	Wang et al. (2022b)
Shenqi Yizhi granule	(1) Modulate synaptic transmission, signal transduction, and amino acid metabolism processes (2) Inhibit response of astrocytes regulated by the JAK2/STAT3 signaling pathway (3) Regulate lipid metabolism, metal ion metabolism, interleukin signaling pathways, and neurotransmitter receptor signaling	Not studied yet	Mice; Rats	An et al. (2018), Ren et al. (2020), Li et al. (2021a), Wang et al. (2022c)
Cordyceps militaris extracts and cordycepin	(1) Increase the expression level of 5-hydroxymethylcytosine and downregulated the transcription level of Apolipoprotein E (2) Downregulate oxidative stress biomarkers and inflammatory cytokines	(1) Modulate gut microbiota to a state with a higher abundance of *Firmicutes/Bacteroidetes* (2) Change the gut microbial composition and increased the levels of acetate and butyrate	Pig	Cao et al. (2020a), Liu et al. (2023)
Bushen-Huatan-Yizhi formula	Affect GSK-3β/CREB signaling pathway	Increase the abundance of *Proteus* and actinomycetes, *Clostridium*, Lactococcus	Rats; Mice	Yang et al. (2021), Cui et al. (2023)
Huanglian Jiedu decoction	(1) Regulate the levels of inflammatory factors (2) Improve abnormal sphingolipid metabolism	(1) Decrease the population of *Firmicutes* and increase the population of *Proteobacteria* at the phylum level (2) Increase the relative abundances of Prevotellaceae, Lactobacillaceae, Peptococcaceae, reduce the relative abundances of Bacteroidales_S24-7_group, Lachnospiraceae and Porphyromonadaceae at the family level	Rats; mice	Gu et al. (2018), Gu et al. (2021), Qi et al. (2022)
SuanZaoRen Decoction	Alleviate Aβ deposit, neuronal loss, synaptic damage and iron accumulation by regulating the DJ-1/Nrf2 signaling pathway	Reverse the altered relative proportions of gut microbiota by decreasing the abundance of *Bacteroidetes* while increasing the *Firmicutes* phylum members	Mice	Du et al. (2023), Long et al. (2024)
*Centella asiatica*	A therapeutic effect on the fear memory deficit and synaptic dysfunction	Modulate the composition of gut microbiota by decreasing harmful genera, such as *Staphylococcus*	Mice	Sukketsiri et al. (2019), Li et al. (2021b)
Naoxintong capsule	Regulate inflammatory cytokines, thereby decreasing p-Tau and Aβ accumulation, inflammation and neuronal apoptosis	(1) Increase the diversity of gut microbiota, (2)Influence the microbiota composition (2) Reverse the increase of the ratio of the *Firmicutes* to *Bacteroidetes* in relative abundance	Mice/Bama Minipig	Zhang et al. (2019), Wang et al. (2021a), Lu et al. (2022a)
Kai-Xin-San	(1) Regulate SIRT3-mediated neuronal cell apoptosis (2) activate the Wnt/beta-catenin signaling pathway (3) inhibit Tau protein hyperphosphorylation, inflammation, and apoptosis	Significantly reverse the decreased abundance of *Bifidobacterium* and *Helicobacter*	Mice/Rats	Jiao et al. (2022), Shan et al. (2023), Su et al. (2023), Wang et al. (2023), Xu et al. (2023)
CZ2HF	Reduce TNF-α, IL-1β, COX-2 protein expression levels, Bax/Bcl-2 ratio, Aβ1-42 and active-caspase-3 levels, as well as IκB-α degradation and p-NF-κB p65 activation	Not studied yet	Rats	Zeng et al. (2019), Wei et al. (2021)
Morinda Officinalis	(1) Regulate the metabolism of amyloid-β (2) enhance the expression of neurotrophic factors (3) inhibit neuroinflammation (4) elevate synapse structure relevant protein	Increase the abundance of *Lactobacillus*, Allobaculum, Lactobacillaceae, and Lachnospiraceae	mice	Cai et al. (2017), Xin et al. (2018)
Huperzine A	Inhibit acetylcholinesterase Wnt signaling, downregulation of activity of GSK-3β, upregulation of Bcl-2 and downregulation of Bax, upregulation of neurotrophin	Not studied yet		Friedli and Inestrosa (2021)
GuanXinNing Tablet (containing CX and DS)	(1) Enhance neuronal metabolism activities (2) Inhibit oxidative stress and neuronal apoptosis	(1) Decrease Firmicutes/Bacteroidetes ratio while improving the abundances of Akkermansia and dgA-11_gut_group (2) DS:decrease Corynebacterium, Blautia, Rhodococcus, Allobaculum while increase Alloprevotella, *Bacteroides*, *Lactobacillus*	Rabbits; rats	Zhang et al. (2021b), Zhang et al. (2021c), Meng et al. (2021), Yin et al. (2024)
Gastrodia elata Blume	(1) P-HBA: alleviate Aβ-induced toxicity via enhancing antioxidant and anti-aging activity and inhibiting Aβ aggregation,anti-oxidative stress (2) Gastrodin: increasing the level of Bcl-2 and decreasing level of Bax, increasing the expression levels of IGF-1 and CREB	GEP-1: modulate gut microbiota by obviously promoting the growth of *Akkermansia muciniphila* and *Lacticaseibacillus paracasei* Gastrodin: positive link with *Firmicutes* and had a negative link with *Cyanobacteria, Proteobacteria,* and *Deferribaceters*		Huo et al. (2021), Fasina et al. (2022), Zhao et al. (2022), Gong et al. (2023), Yu et al. (2024)

Secondly, up to now, many herbs or herbal preparations have been proven to have modulatory effects on gut microbiota, while there are still no relevant studies elucidating possible neuroprotective effects on AD models via modulating gut microbiota. At the same time, some herbs have been proven to be beneficial for AD models *in vitro* and be possible drug candidates for AD, but there is a lack of up-to-date literature demonstrating their potential effects on gut microbiota. In this case, we reviewed some examples of herbal medicine from Traditional Chinese Medicine, European Medicine, Indian Medicine, Thai Medicine, and summarized the present evidence on their connections with AD and whether they have an influence on the gut microbiota.

### 4.2 Herbs in traditional Chinese medicine

Here we discuss some examples of individual herbs, compounds or extracts from herbs and herbal formulae from Traditional Chinese Medicine and their effects on gut microbiota, which are shown in Table 2 and Table 3.

**TABLE 2:** Examples of herbs or compounds/extracts from herbs and their effects on gut microbiota in Traditional Chinese Medicine.

Herbs or compounds/extracts from herbs	Herbs	Effects on gut microbiota	Models	References
Ponicidin	Rabdosia rubescens	Restore the relative abundance of *Allobaculum*, *Lactobacillus* and *Ruminococcus* genera	Rats	Zhang et al. (2023c)
Radix Scrophulariae	Radix Scrophulariae	Upregulate the number of *Lactobacillus* species, elevate the level of some metabolites from gut microbiota, such as Guanosine, hypoxanthine, xanthine and inosine levels	Rats	Lu et al. (2019)
LB	LB	Rectify the disturbed floras including *Porphyromonadaceae, Lactobacillus* and *Escherichia* at the genus level	Rats	Bubu et al. (2017), Si et al. (2022)
6-gingerol	Zingiber officinale Roscoe	Increase the relative abundance of beneficial *Bacteroidetes* and decrease *Firmicutes* on the phylum level	Rats	Lai et al. (2022)
WMP	walnut kernel	Reduce the abundance of *Fusobacterium varium* and *Bacteroides vulgatus*, increase the abundance of *Lactobacillus animalis*	Rats	He et al. (2022)
GPM extract	Gynostemma pentaphyllum	(1)Decrease the ratio of *Firmicutes* to *Bacteroidetes* (2) Increase the relative abundance of some beneficial bacteria, such as *Butyricimonas*, *Lactobacillus* and reduced the relative abundance of pathogenic bacteria including *Bilophila*, *Blautia* and *Dorea*	Mice or *in vitro* studies	Wang et al. (2022d), Shu et al. (2022)
Andrographolide	Andrographis paniculata	Increase the abundance of beneficial microbiota, such as *the f__Lachnospiraceae_Unclassified, Lachnospiraceae_ NK4A136_group* and *Ruminococcaceae_ UCG-014*	Mice	Wu et al. (2021)
Triptolide	Tripterygium Wilfordii Hook F	Rectify the gut microbiota by decreasing the population of *Bacteroidetes, Bacteroides* and *Lachnospiraceae* along with increasing that of *Firmicutes*	Mice	Wu et al. (2019)
PCA	Duzhong	Increase the ratio of *Firmicutes* to *Bacteroidetes* by increasing the relative abundance of *Firmicutes* and inhibiting *Bacteroidetes*	Piglets	Hu et al. (2020)
PLS	Pueraria lobata (Willd.) Ohwi	Increase the *Firmicutes*, decrease the level of *Proteobacteria*. Promote growth of *Lactobacillus* while inhibit *Escherichia*-*Shigella*, *Enterococcus* and *Desulfovibrio* at genus level	Mice	Yang et al. (2022)
Salidroside	Rhodiola rosea L	Increase Alloprevotella and Parasutterella and decrease the abundance of Prevotellaceae and Ruminiclostridium	Mice	Olennikov et al. (2020)
DJP	D. loddigesii	Ameliorate gut microbiota disturbance by increasing the *Bacteroidetes* to *Firmicutes* ratio and the relative abundance of *Prevotella*/*Akkermansia*, while reducing the relative abundance of *Escherichia* coli	Mice	Li et al. (2018)

WMP, extracts from walnut meal; PCA, protocatechuic acid; PLS, P. lobata starch; LB, Lilium brownii F. E. brown; DJP, rich-polyphenols extract of D. loddigesii.

**TABLE 3:** Examples of herb pairs or herbal formula/preparations and their effects on gut microbiota in Traditional Chinese Medicine.

Herb pair or herbal formula/preparations	Effects on gut microbiota	Models	References
Radix ginseng and Schisandra chinensis	Regulate gut microbiota and attenuate the elevated metabolites of p-cresol	Rats	Wang et al. (2021b), Wang et al. (2022e)
Qisheng Wan formula	Elevate the abundance of the *Lactobacillus* genus and decrease the relative abundance of *Alistipes* and the *Lachnospiraceae NK4A136* group	Rats	Xiong et al. (2022)
WangShiBoChiWan	Modulate gut microbiota community by increasing *Lactobacillus* genus, decreasing *Bacteroides fragillis* group	Mice	Yin et al. (2021)
Wumei decoction or pills	Regulate gut microbiota dysbiosis by increasing *Allobaculum* and *Bacteroides* while decreasing *Ileibacterium*	Mice	

#### 4.2.1 Rabdosia rubescens and ponicidin

Ponicidin is a diterpenoid isolated from a Chinese traditional herb Rabdosia rubescens. As diabetes mellitus is an important factor contributing to cognitive impairment by increasing the production of free radicals and enhancing inflammatory reactions, there is evidence supporting the neuroprotective role of ponicidin in alleviating diabetic cognitive dysfunction a in rat model (Zhang X. et al., 2023). This study showed that ponicidin significantly attenuated cognitive impairment not only through alleviating inflammation and oxidative stress, but also through modulating gut microbiota. To be specific, ponicidin significantly decreased the level of pro-inflammatory factors and increased the level of anti-inflammatory factors in the brain and the serum. Ponicidin can effectually restore the relative abundance of *Allobaculum*, *Lactobacillus* and *Ruminococcus* genera, which participate in alleviating diabetic cognitive impairment. However, there is still no direct evidence supporting the neuroprotective role of ponicidin in AD models by regulating gut microbiota composition.

#### 4.2.2 Radix Scrophulariae (RS)

Radix Scrophulariae, the dried root of Scrophularia ningpoensis Hemsl., belongs to the Scrophulariaceae family. Recent research indicated that Radix Scrophulariae altered the structure and composition of the gut microbiota and substantially altered the gut metabolic phenotype. It was shown that RS upregulated the number of *Lactobacillus* species present in the gut of the rat and elevated the level of some metabolites from gut microbiota, such as guanosine, hypoxanthine, xanthine, and inosine in rats (Lu et al., 2019). RS may provide beneficial effects on AD patients through regulating the composition of gut microbiota (Wei, 2016).

#### 4.2.3 Lilium brownii F. E. Brown

In clinical practice of Traditional Chinese Medicine, Lilium Brownie F. E. Brown (LB) is a Chinese herb commonly used to relieve insomnia which lasts for a long time. A comprehensive study illustrates that LB may relieve insomnia through rectifying the disturbance of gut microbiota and related metabolites. 16S rRNA gene sequencing demonstrated that LB rectified the disturbed floras including *Porphyromonadaceae, Lactobacillus* and *Escherichia* at the genus level (Si et al., 2022). From another perspective, previous studies have indicated the intensely adverse influence of insomnia on cognitive functions of AD patients (Bubu et al., 2017; Pak et al., 2020; Yoon et al., 2023). Therefore, LB may ameliorate impaired cognitive functions of AD patients directly through maintaining the gut microbiota homeostasis or indirectly through alleviation of sleep problems.

#### 4.2.4 Zingiber officinale Roscoe and 6-gingerol

Zingiber officinale Roscoe, also known as ginger, is a widely used medicinal herb in Traditional Chinese Medicine. Increasing studies have proven that ginger possesses a variety of beneficial effects, such as anti-inflammatory, anti-oxidant, and neuroprotective properties (Luo et al., 2021; Upadhyaya et al., 2022; Hu et al., 2023). Therefore, it can be a potential agent in protecting against neurodegenerative diseases, such as AD (Talebi et al., 2021; Arcusa et al., 2022). More and more evidence has manifested that ginger can increase the relative abundance of beneficial *Bacteroidetes* and decrease *Firmicutes* on the phylum level in rats, showing prebiotic-like properties. As there is a high concentration of polyphenols, mainly 6-gingerol, in ginger, this may be responsible for the prebiotic-like effects (Lai et al., 2022). It is reasonable to speculate that the modulatory effect of ginger on the richness, diversity and composition of gut microbiota is also an essential part of the protective role of ginger in ameliorating cognitive impairment and AD. Additional studies still need to be conducted to further elaborate on this speculation.

#### 4.2.5 Walnut meal extracts

Walnut meal is a by-product derived from the cold pressed walnut kernel, a traditional Chinese herb. It was reported that extracts from walnut meal (WMP) restored the diversity of gut microbiota which was reduced by high-fat diet through reducing the abundance of Gram-negative bacteria, such as *Fusobacterium varium* and *Bacteroides vulgatus*, and increasing the abundance of *Lactobacillus animalis* (He et al., 2022). Existing studies have indicated that chronic intake of a high-fat diet is related to cognitive dysfunction by inducing alterations in the density of neurotransmitter receptors in brain regions associated with cognition, as well as to gut microbiota dysbiosis (Amelianchik et al., 2022; Gannon et al., 2022). Therefore, it is reasonable to speculate that extracts from walnut meal can be a protective agent against AD by maintaining gut microbiota homeostasis in the context of a high-fat diet. Specifically, WMP greatly decreased the abundance of *Bacteroides* vulgatus, and significantly increased the abundance of *Lactobacillus*. As walnut meal extracts consist of polyphenols, the modulatory effect of walnut meal extracts on gut microbiota composition may be mainly attributed to polyphenols.

#### 4.2.6 Gynostemma pentaphyllum and its compounds

Gynostemma pentaphyllum (GPM), also called Jiaogulan, is a traditional Chinese medicinal herb and contains more than 230 compounds that have been discovered, mainly saponins, as well as polysaccharides and flavonoids. These compounds have been shown to possess diverse pharmacological properties, including anti-inflammatory, antioxidant, and immune-modulatory activities. Also, a review summarized the prebiotic properties of these compounds, showing the therapeutic potential of Jiaogulan on the diversity and composition of gut microbiota (Huang et al., 2022). Jiaogulan promotes the growth of beneficial bacteria, particularly the short-chain fatty acid (SCFA) producers. GPM water extract (GPWE) and GP-IV (a saponin monomer in GPM) decreased the ratio of *Firmicutes* to *Bacteroidetes*, increased the relative abundance of several benefcial bacteria, such as *Butyricimonas*, *Lactobacillus,* and reduced the relative abundance of pathogenic bacteria including *Bilophila*, *Blautia* and *Dorea* that were associated with metabolic disorders (Shu et al., 2022). A large number of studies reveal that these bioactive compounds can ameliorate memory impairment of AD models *in vitro* or *in vivo*. Take Gypenoside as an example, a major bioactive component of GPM, could suppress microglial activation when exposed to Aβ by maintaining the microglial M1/M2 state as a result of suppressing the expression of cell signaling protein 1 (SOCS1) in N9 microglial cells (Cai et al., 2016). Gypenoside can alleviate LPS-induced neuroinflammation and memory impairment in rats by decreasing the levels of proinflammatory mediators as well as suppressing levels of inducible nitric oxide synthase (iNOS), toll-like receptor 4 (TLR4) and inhibition of brain-derived neurotrophic factor (BDNF) mRNA (Lee et al., 2018). In network pharmacology analysis, the primary targets of active compounds from GPM include epidermal growth factor receptor (EGFR), interleukin-1 beta (IL-1β), interleukin-6 (IL-6), nitric oxide synthase in endothelial (NOS3), and the neuroprotective mechanisms are connected with HIF-1 signaling pathway, cytokine-cytokine receptor interaction (Wang et al., 2022d). Gypenoside LXXV (GP-75), a novel component isolated from GPM, could ameliorate cognitive deficits in a mouse model of diabetic AD (APP/PS1xdb/db mice) by enhancing glucose uptake through activation of specific signaling pathways (Meng et al., 2023). However, there is no direct evidence suggesting the modulatory effect of these compounds on microbiota in AD models.

#### 4.2.7 Andrographis paniculata and andrographolide

Andrographolide is a compound isolated from the Chinese herb Andrographis paniculata. Animal studies found that andrographolide can directly influence the composition of gut microbiota by increasing the proportion and the abundance of beneficial microbiota, such as *f__Lachnospiraceae_ Unclassified, Lachnospiraceae_ NK4A136_group* and *Ruminococcaceae_ UCG-014*. Meanwhile, the composition of gut microbiota was dominated by *Firmicutes* after andrographolide treatment (Wu et al., 2021), indicating that andrographolide can have a role in restoring gut microbiota balance and may exert possible therapeutic effects on AD models, which also warrants more research to provide more evidence.

#### 4.2.8 Tripterygium Wilfordii Hook F

Although the clinical application of Tripterygium Wilfordii Hook F is strictly limited because of its severe toxicity, triptolide, an active component extracted from this traditional Chinese herb, has already been proven to possess immunosuppressive and anti-inflammatory properties. Existing studies revealed that triptolide could regulate the function of immune cells and the expression of cytokines through inflammatory signaling pathways, thus exerting potent therapeutic effects (Cheng et al., 2021). In the meantime, it was shown to play a role in maintaining gut microbiota homeostasis. It was reported that administration of triptolide in mice could restore the biological diversity and composition of gut microbiota through decreasing the population of *Bacteroidetes, Bacteroides* and *Lachnospiraceae,* as well as increasing that of *Firmicutes* (Wu et al., 2019), and the therapeutic effects of triptolide was related to its regulatory role in restoring the gut microbiota. In addition, a recent study demonstrated that gut microbiota exerted protective effects on triptolide-induced liver injury (Huang et al., 2020). We can assume that triptolide may be beneficial in protecting against AD by regulating the gut microbiota, which may be a novel target for further investigation.

#### 4.2.9 Eucommia ulmoides Oliver

Eucommia ulmoides Oliver, also known as Duzhong, is a Chinese herb containing an active phenolic acid called protocatechuic acid (PCA). Protocatechuic acid is also a bioactive metabolite of polyphenols against oxidative stress and inflammation. PCA significantly increased the ratio of *Firmicutes* to *Bacteroidetes* by increasing the relative abundance of Firmicutes and inhibiting Bacteroidetes in LPS-challenged piglets (Hu et al., 2020). At the genus level, PCA significantly decreased the relative abundance of *Prevotella 9, Prevotella 2, Runminococcus* torques group and *Holdemanella*, which were all positively correlated with the production of inflammatory cytokines, or negatively correlated with the expression of tight junction proteins. Meanwhile, PCA significantly increased the relative abundance of *Roseburia* and *Desulfovibrio*, which were negatively correlated with the inflammatory markers, or positively correlated with the expression of tight junction proteins.

#### 4.2.10 Pueraria lobata (Willd.) Ohwi and P. lobata starch

Pueraria lobata (Willd.) Ohwi is an edible Chinese medicinal herb, the dried roots of which are enriched in starch called P. lobata starch (PLS). It is well-established that resistant starch belongs to polysaccharides that can be utilized by gut microbiota, which is accompanied by an increase in SCFAs in the gut and blood circulation. The effect of PLS on gut microbiota composition was assessed *in vitro* and *in vivo*. Evidence showed that *in vitro* fermentation of PLS can significantly increase the number of *Firmicutes* and decrease the number of *Proteobacteria*. Furthermore, at the genus level, it can significantly promote the growth of *Lactobacillus* and inhibit the growth of *Escherichia*-*Shigella*, *Enterococcus* and *Desulfovibrio* (Yang et al., 2022). It seems that PLS takes a potential prebiotic effect on the composition of gut microbiota. Furthermore, PLS can attenuate gut dysbiosis induced by HFD in mice, specifically, it can increase *Firmicutes* and *Desulfobacterota*, and decrease Bacteroidota at the phylum level. The potential prebiotic effect of PLS makes it a good candidate for prevention and treatment of AD.

#### 4.2.11 Rhodiola rosea L. and Salidroside

Rhodiola rosea L. is a renowned medical plant which contains abundant and various compounds with antioxidant properties, not only in roots or rhizomes, but also in leaves, flowers and stems. This makes it a potential plant for producing new bioactive and functional products for clinical application. Salidroside (SAL), a phenylpropanoid compound, is derived from rhizomes and roots of this plant. Notably, administration of SAL in mouse models for age-related AD could increase the abundance of *Alloprevotella* and *Parasutterella* and decrease the abundance of *Prevotellaceae* and *Ruminiclostridium* (Olennikov et al., 2020). In addition, SAL was able to restore the integrity of the intestinal barrier, to decrease the level of proinflammatory cytokines that are involved in cognitive impairment or AD pathology, as well as to reduce toxic Aβ peptide deposition accompanied by a reduction in microglial mediated neuroinflammation (Xie et al., 2020). The beneficial effect of SAL on cognitive function may be ascribed to, in part, the recovery of the composition of the gut microbiota.

#### 4.2.12 Dendrobium and its extracts

Dendrobium is a traditional Chinese herb that contains plenty of compounds, such as polyphenols, polysaccharides, and alkaloids (Guo et al., 2020). D. loddigesii is one of the common Dendrobium crude drugs in Traditional Chinese Medicine. The polyphenol-rich extract of this herb showed anti-inflammatory and antioxidant activities (Chen et al., 2018). Previous research has already demonstrated that polysaccharides from Dendrobium alleviated learning and memory impairment in animal models through its antioxidant and anti-inflammatory actions (Liang et al., 2019; Li et al., 2020). *In vivo* treatment with a polyphenol-rich extract of D. loddigesii (DJP) in mice showed anti-inflammatory effects, by decreasing the expression of IL-6 and TNF-α, as well as ameliorating gut microbiota disturbance, by increasing the *Bacteroidetes* to *Firmicutes* ratio and the relative abundance of *Prevotella*/*Akkermansia*, and reducing the relative abundance of *Escherichia* coli (Li et al., 2018). Combined together, the protective effect of Dendrobium on memory impairment may be partly attributed to the modulatory effect on the composition of gut microbiota.

#### 4.2.13 Radix ginseng and schisandra chinensis (GS)

In Traditional Chinese Medicine, herb pairs are common prescriptions seen in clinical settings, which refer to the unique combinations of two relatively constant herbs. One of these pairs is the compatibility of Radix ginseng and GS, which have been widely used to improve cognitive dysfunction in the clinical practice of the Traditional Chinese Medicine. It has been reported that the beneficial effects of the herb pair of GS on AD were attributed to their capability of regulating metabolic processes, such as bile acid biosynthesis, sphingolipid metabolism, porphyrin and chlorophyll metabolism (Wang A. et al., 2022). Changes in the gut microbiota lead to the overgrowth of specific p-cresol-producing bacteria, and GS attenuated the elevated metabolites of p-cresol by gut microbiota in AD rats, suggesting that GS has a therapeutic effect on gut microbiota dysbiosis observed in AD (Wang A. et al., 2021).

#### 4.2.14 Qisheng Wan formula

Qisheng Wan formula (QWF) is a classic Chinese formulation containing seven herbal drugs and widely used to treat patients with dementia, which is recorded in Taiping Huimin Heji Ju Fang. A recent study proved that QWF was effective in managing AD and one of the therapeutic mechanisms is to modulate the diversity and composition of gut microbiota in animal models by elevating the abundance of *Lactobacillus* genus and decreasing the relative abundance of *Alistipes* and the *Lachnospiraceae NK4A136* group in AD rats (Xiong et al., 2022).

#### 4.2.15 WangShiBoChiWan

WangShiBoChiWan (WSBCW) is a traditional Chinese medicine which includes Radix et Rhizoma Rhei, Rhizoma coptidis, Rhizoma Arisaematis, Buibus Fritillariae cirrhosae, and Fructus Crotonis. Preclinical evidence demonstrated the beneficial effects of WSBCW on gastrointestinal health, as it can not only modulate intestinal permeability, but also the gut microbiota by increasing bacteria in the *Lactobacillus* genus, decreasing those of the *Bacteroides fragillis* group, while not having significant effects on the abundance of *Clostridium coccoides*, *Clostridium leptum*, or *Prevotella* group (Yin et al., 2021). There is still a lack of relevant studies on the preventive and therapeutic effects of this medicine on AD targeting the composition of gut microbiota.

#### 4.2.16 Wumei decoction or pills

Wumei decoction or pills, a classical formula described in Shanghan Lun, could significantly reduce the secretion of proinflammatory cytokines, maintain the epithelial barrier function and regulate gut microbiota dysbiosis by increasing *Allobaculum* and *Bacteroides* while decreasing *Ileibacterium* in mouse models of chronic colitis, thereby exerting anti-inflammatory effects (Lu DX. et al., 2022; Wu et al., 2022; Duan et al., 2023). A meta-analysis supported the efficacy and advantages of Wumei bolus in reducing inflammatory factors, improving symptoms in patients, and providing basic evidence for clinical application of Wumei prescription (Chen et al., 2023). However, it is still unknown whether this regulatory effect on gut microbiota by Wumei exerts beneficial roles in AD models and needs to be further investigated.

### 4.3 European medicine

#### 4.3.1 Tiliae flos

In European Medicine, the linden flower (Tiliae flos) is a herb which has been used for centuries. Studies showed that raw linden flower extracts contained mainly flavan-3-ols and flavonoids, such as quercetin and kaempferol derivatives, and the original composition of the extracts was changed by the human gut microbiota. After biotransformation of the extracts, they exert a beneficial influence on human gut microbiota by changing the composition of the gut microbiota, like increasing the abundance of bacteria belonging to the *Succinivibrionaceae, Lachnospiraceae, Clostridiaceae, Ruminococcaceae,* and *Eubacteriaceae* families. That is, increasing the abundance of bacteria responsible for SCFAs production and exerting an anti-inflammatory effect (Kruk et al., 2022). In addition, its bioavailable metabolites exhibit direct activity of inhibiting the production of cytokines. Further investigations need to be conducted to verify whether the anti-inflammatory effect of the extracts is protective against AD.

#### 4.3.2 Artemisia absinthium L

Artemisia absinthium L, known as a medicinal plant in Europe, has some components which have been confirmed to be able to exert anti-inflammatory, neuroprotective effects by previous studies. Caruifolin D from Artemisia absinthium L. could inhibit neuroinflammatory responses by decreasing the expression of iNOS and COX-2 in LPS-treated BV-2 cells (Zeng et al., 2015) and it might be applied to treat neuroinflammation-related diseases. By far, there is still no relevant literature demonstrating the influence of Caruifolin D on gut micoriota. However, other components like phenolic acids, flavonoids, and polysaccharides from Artemisia absinthium L. possess antioxidant, immuno-modulatory activities and microbiota-modulatory effects. The bioactive compounds of Artemisia absinthium and their pharmacological activities as well as relevant mechanisms have been reviewed by a number of studies (Batiha et al., 2020; Szopa et al., 2020; Sharifi-Rad et al., 2022).

### 4.4 Ayurveda and relevant herbs

#### 4.4.1 Ulmus rubra and Glycyrrhiza glabra

The traditional medicine in India is called Ayurveda. It is shown that Ulmus rubra (common name: slippery elm) and Glycyrrhiza glabra (common name: licorice), as well as a polyherbal medicinal formulation (common name: triphala) have a prebiotic potential and are capable of modulating the gut microbiota (Peterson et al., 2018), which may at least in part explain the beneficial effects of these herbs on gastrointestinal health. The prebiotic role of these herbs on AD models or patients still needs to be confirmed in animal studies or clinical trials.

#### 4.4.2 Nervine herbal medicines in Ayurveda

The commonly used medicinal herbs to treat neurodegenerative diseases, such as AD, in Ayurveda are called nervine herbal medicines, including Kapikacchu, Bacopa, Shankhapushpi, Frankincense, Jatamansi, Bhringaraj, Guduchi, Ashwagandha and Shatavari. Recent work has established the prebiotic property of these medicinal herbs which provide diverse substrates for gut microbes to utilize, thererafter altering the composition of the gut microbiota (Peterson et al., 2019). The prebiotic effect of these medicinal herbs on the composition of the gut microbiota may partly explain their protective influence on AD models or patients. However, the lack of direct evidence warrants further investigations. The medicinal herb-driven prebiotic effects lead to changes in metabolism and generation of specific metabolites by the gut microbiota which can bring benefits and therapeutic effects. Bacopa monnieri, a herb that has been used for many centuries in India, has shown neuroprotective effects in animal and in *in vitro* studies. A recent systematic review yielded 5 eligible human studies which used B. monnieri to treat patients with AD and provided that there was no difference between B. monnieri and the placebo or donepezil in the treatment of AD (Basheer et al., 2022) B. monnieri contains diverse bioactive components belonging to alkaloids, saponins, flavonoids, triterpenes and cucurbitacin, which were proven to be neuroprotective (Fatima et al., 2022). In animal AD models, it was found that extract of B. monnieri significantly attenuated neuronal loss and restored cholinergic neuron densities (Uabundit et al., 2010), decreased the level of amyloidogenic proteins by up to 60% in the brain (Holcomb et al., 2006). An *in vitro* study found that this extract reduced the amount of amyloid fibrils and dismantled the preformed amyloid fibrils. Meanwhile, an *in silico* study showed that two saponins from B. monnieri exerted a favorable binding affinity with the Caspase-3 and tau-protein kinase I (TPK I) receptors that are therapeutic targets of AD (Bishbishy et al., 2019). These findings demonstrated the therapeutic potential of B. monnieri for AD.

#### 4.4.3 Justicia secunda Vahl

Extracts from Justicia secunda Vahl, a traditional medicinal plant in India, include flavonoids, phenolic acids, and alkaloids (Swiatek et al., 2023). Some extracts could exert neuroprotective properties by inhibiting activities of the acetylcholinesterase (AChE) and the butyrylcholinesterase (BChE) (Oboh et al., 2013), indicating its potential application to manage AD. A recent study revealed that lignan glycosides isolated from Justicia secunda Vahl exhibited antimicrobial properties like moderate antibacterial activities against *Staphylococcus aureus* SA RN 46003 (Kamlo et al., 2023). Functional tea made from fresh leaves of Justicia secunda is rich in health-supporting substances like flavonoids and alkaloids which are beneficial to the body (Uchegbu et al., 2023). There is a lack of scientific literatures demonstrating the effect of Justicia secunda Vahl. and its extracts on gut microbiota.

#### 4.4.4 Acalypha indica

Acalypha indica is an Indian copperleaf plant and methanolic extracts of the root are rich in phenols and flavonoids. These extracts showed anti-inflammatory properties by decreasing hydroxyl radicals, lipid peroxidation *in vitro* and activities of the superoxide dismutase, glutathione peroxidase in rats (Sahukari et al., 2021). Based on biochemical and molecular docking methods, extracts from Acalypha indica inhibited harmful bacteria strains, mainly *Streptococcus* iniae and *Staphylococcus* sciuri (Shimu et al., 2023). It is possible that this antibacterial property can have an impact on neuroinflammatory diseases like AD, which needs to be further validated by future studies.

### 4.5 Thai medicine

#### 4.5.1 The Kleeb Bua Daeng formula

The Kleeb Bua Daeng formula (KBD), a Thai traditional medicine with multi-target activities could inhibit activities of the AChE, Aβ aggregation, the expression of pro-apoptotic factors in an *in vitro* study (Chheng et al., 2020)and show protective effects on memory impairment in animals (Waiwut et al., 2022). A phase I randomized, double-blind, placebo-controlled trial provided clinical evidence supporting the efficacy and safety of the Kleeb Bua Daeng formula in mild cognitive impairment patients by demonstrating the improved cognitive performance compared to the baseline though there was no significant difference between KBD and the placebo groups (Musigavong et al., 2022). The effect on gut microbiota by the Kleeb Bua Daeng formula has not been elucidated yet. Examples of herbal medicine that modulate the gut microbiota in European medicine, Indian medicine, Thai medicine are illustrated in Table 4.

**TABLE 4:** Examples of herbal medicine that exhibit neuroprotective properties on AD models, and their possible effects on gut microbiota in European medicine, Indian medicine, Thai medicine.

Herbs or extracts	Country	Neuroprotective properties on AD or effects on gut microbiota	Models	References
Tiliae flos	Europe	Increase the abundance of bacteria responsible for SCFAs production, and bacteria belonging to the *Succinivibrionaceae, Lachnospiraceae, Clostridiaceae, Ruminococcaceae,* and *Eubacteriaceae* families	Human gut microbiota *ex vivo*	Kruk et al. (2022)
Artemisia absinthium L	Europe	Decrease iNOS and COX-2 expressions	BV-2 cells	Zeng et al. (2015)
Ulmus rubra, Glycyrrhiza glabra Triphala	India	A prebiotic potential and capable of modulating the gut microbiota	*In vitro* study	Peterson et al. (2018)
Kapikacchu, Bacopa Shankhapushpi, Frankincense,Jatamansi, Bhringaraj,Guduchi, Ashwagandha	India	Alter the composition of gut microbiota through their prebiotic properties	*In vitro* study	Peterson et al. (2019)
Justicia secunda Vahl	India	Exhibit antimicrobial properties like moderate antibacterial activity against *Staphylococcus aureus* SA RN 46003	*In vitro* study	Kamlo et al. (2023)
Acalypha indica	India	Inhibit harmful bacteria strains, mainly *Streptococcus iniae* and *Staphylococcus sciuri*	*In vitro* study	Shimu et al. (2023)
Kleeb Bua Daeng formula	Thai medicine	Inhibit activities of the AChE, Aβ aggregation, expression of pro-apoptotic factors, mitigate memory impairment	*In vitro* study; animals	Waiwut et al. (2022), Ospondpant et al. (2023)
Dracaena cochinchinensis	Thailand	Suppress Aβ1-42 fibril formation, clear Aβ aggregation; decrease expression of pro-inflammatory factors, while increase expression of the anti-inflammatory biomarker, as well as alleviate excessive phagocytosis of Aβfibrils	In cultured PC12 cells; in both BV2 microglia and RAW264.7 macrophage	Shiao et al. (2017), Ospondpant et al. (2023)
Acanthus ebracteatus	Thailand	Possess antimicrobial activity against *E. coli* and *L. monocytogenes* *in vitro* Ameliorating amyloid deposition, cholinergic dysfunction, Aβ 1-40 production, Aβ 1-42 oligomerization	Aβ_1-42_- rats; *In vitro* SH-SY5Y cells	Anukulthanakorn et al. (2016), Shiao et al. (2017)
Pueraria mirifica	Thailand	Downregulate the Bace1 mRNA level and App mRNA level; Reduce NO production and iNOS expression	In rats; microglial cell line	Anukulthanakorn et al. (2016)

#### 4.5.2 Dracaena cochinchinensis

Dracaena cochinchinensis is a Thai folk medicine which has been used in Thai traditional medicine for a long period. Extracts of D.cochinchinensis stemwood displayed pharmacological activities including suppressing Aβ_1-42_ fibril formation, Aβ aggregation, promoting neuronal survival and differentiation in cultured PC12 cells, and these were ascribed to the constituent resveratrol in this medicine (Ospondpant et al., 2022). Another study showed that its extracts exhibited anti-neuroinflammatory properties by decreasing the expression of pro-inflammatory factors and increasing the expression of anti-inflammatory biomarkers in both BV2 microglia and RAW264.7 macrophages, as well as alleviating excessive phagocytosis of Aβfibrils during the LPS-mediated microglial activation (Ospondpant et al., 2023). Although no scientific literatures directly demonstrate the relationship between gut microbiota and D.cochinchinensis, it is reasonable to speculate that D.cochinchinensis may modulate gut microbiota composition via resveratrol present in it.

#### 4.5.3 Acanthus ebracteatus

Acanthus ebracteatus is a plant used as an important ingredient in neuro-tonic remedies for improving brain functions in Thai traditional medicine. The extract from leaves and the stem could inhibit the release of pro-inflammatory cytokines from RAW 264.7 murine macrophages (Wisuitiprot et al., 2022) and possess antimicrobial activities against *E. coli* and *L. monocytogenes in vitro* (Olatunji et al., 2022). Evidence has shown that phytochemical compounds from Acanthus ebracteatus, including Acteoside and isoacteoside, are able to ameliorate cognitive dysfunction, amyloid deposition, and cholinergic dysfunction in Aβ_1–42_- rats, as well as to inhibit Aβ _1–40_ production, Aβ_1–42_ oligomerization and to improve cell viability in SH-SY5Y cells (Shiao et al., 2017). In addition, its leaf extract displayed neuroprotective properties against oxidative stress induced by glutamate (Prasansuklab and Tencomnao, 2018), which supported its potential for treating AD (Prasansuklab et al., 2020).

#### 4.5.4 Pueraria mirifica

Pueraria mirifica is a Thai medicinal plant well known for possessing various pharmacological properties. Pueraria mirifica extracts exhibited neurotherapeutic effects in early- and late-stage cognitively impaired rats by down-regulating the Bace1 mRNA and the App mRNA (Anukulthanakorn et al., 2016). They also reduced NO production and iNOS expression, suppressed mRNA expression of monocyte chemoattractant protein-1 (MCP-1), IL-6, and TNF-α, inhibited microglial activation induced by LPS, exerting anti-inflammatory effects in a rat microglial cell line (Jantaratnotai et al., 2022). The effect of Pueraria mirifica extracts on gut microbiota has not been documented, which may be a novel focus in future studies.

## 5 Relationship between Herbal medicine and AD through modulating the gut microbiota

**FIGURE 4:**
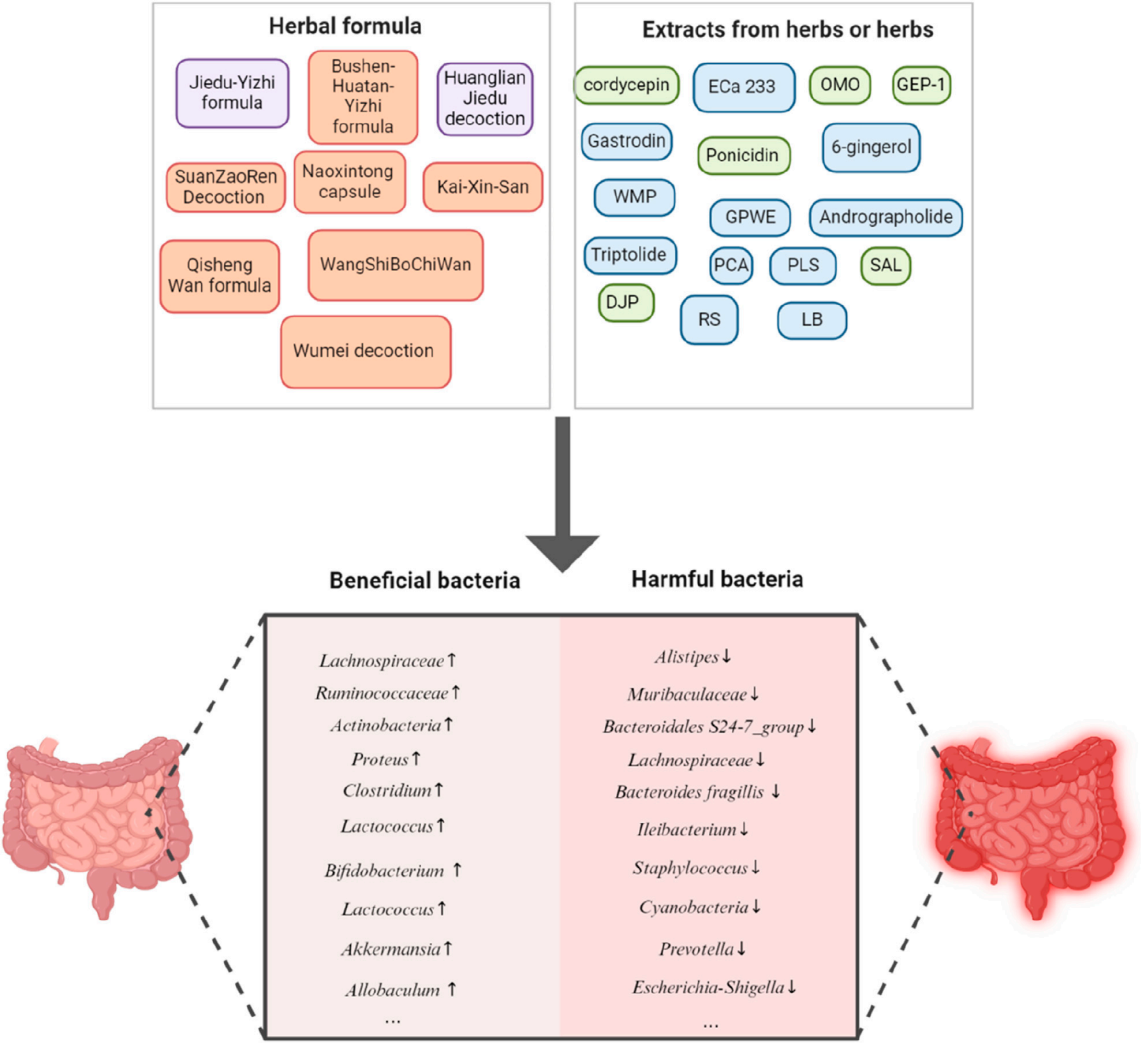
A general summary of the specific herbal formulae, herbal extracts or herbs and their influence on beneficial bacteria or harmful bacteria in our review.The black arrow pointing upwards indicates upregulation, the black arrow pointing downwards indicates downregulation. Cordycepin: extracted from Cordyceps militaris; ECa: extracted from *Centella asiatica*; OMO: Oligosaccharides from Morinda officinalis; GEP-1: a polysaccharide from Gastrodia elata; Gastrodin: an active ingredient from Gastrodia elata; Ponicidin: a diterpenoid from Rabdosia rubescens, 6-gingerol: a polyphenol from Zingiber officinale Roscoe; WMP: extracts from walnut meal; GPWE: Gynostemma pentaphyllum water extract; Andrographolide: a compound isolated from Andrographis paniculata; Triptolide: extracted from Tripterygium Wilfordii Hook F; PCA: Protocatechuic acid, from Eucommia ulmoides Oliver; PLS: P. lobata starch, from Pueraria lobata (Willd.) Ohwi; SAL: Salidroside, a phenylpropanoid from Rhodiola rosea L.; DJP: rich-polyphenols extract of D. loddigesii.; RS: Radix Scrophulariae; LB: Lilium brownii F. E. Brown.

It is reasonable to speculate that the modulatory effect on gut microbiota may also be an important and beneficial aspect of herbal medicine for the prevention and treatment of AD. The specific herbal formulae or herbal extracts mentioned in our review and their influence on beneficial bacteria or harmful bacteria is generally summarized in Figure 4. Much more efforts are still needed in the future to obtain further insights into the relationships between herbal medicine, gut microbiota and AD.

## 6 Discussion

The multi-target beneficial effects of herbs or herbal formulae in the treatment of AD have been extensively elucidated and the associated mechanisms are gradually revealed and validated. More and more evidence also supports the regulatory roles of herbs or herbal formulae in modulating the gut microbiota. After herbs or herbal formulae enter the gut, they are transformed and absorbed into the body, thereby exerting the complicated effects through the circulation system. At the same time, ingredients of herbs or herbal formulae interact with the gut microbiota and to some extent influence the composition of microbiota in the gut. Based on this understanding, it is reasonable to postulate that the direct influence of herbal components on gut microbiota could have secondary effects on the host through the microbiota-gut-brain axis. As interactions between gut microbiota and AD also have been well-documented, the secondary effects exerted by altered gut microbiota may be an essential part of the neuroprotective mechanisms of herbs in ameliorating cognitive impairment in AD.

**FIGURE 5:**
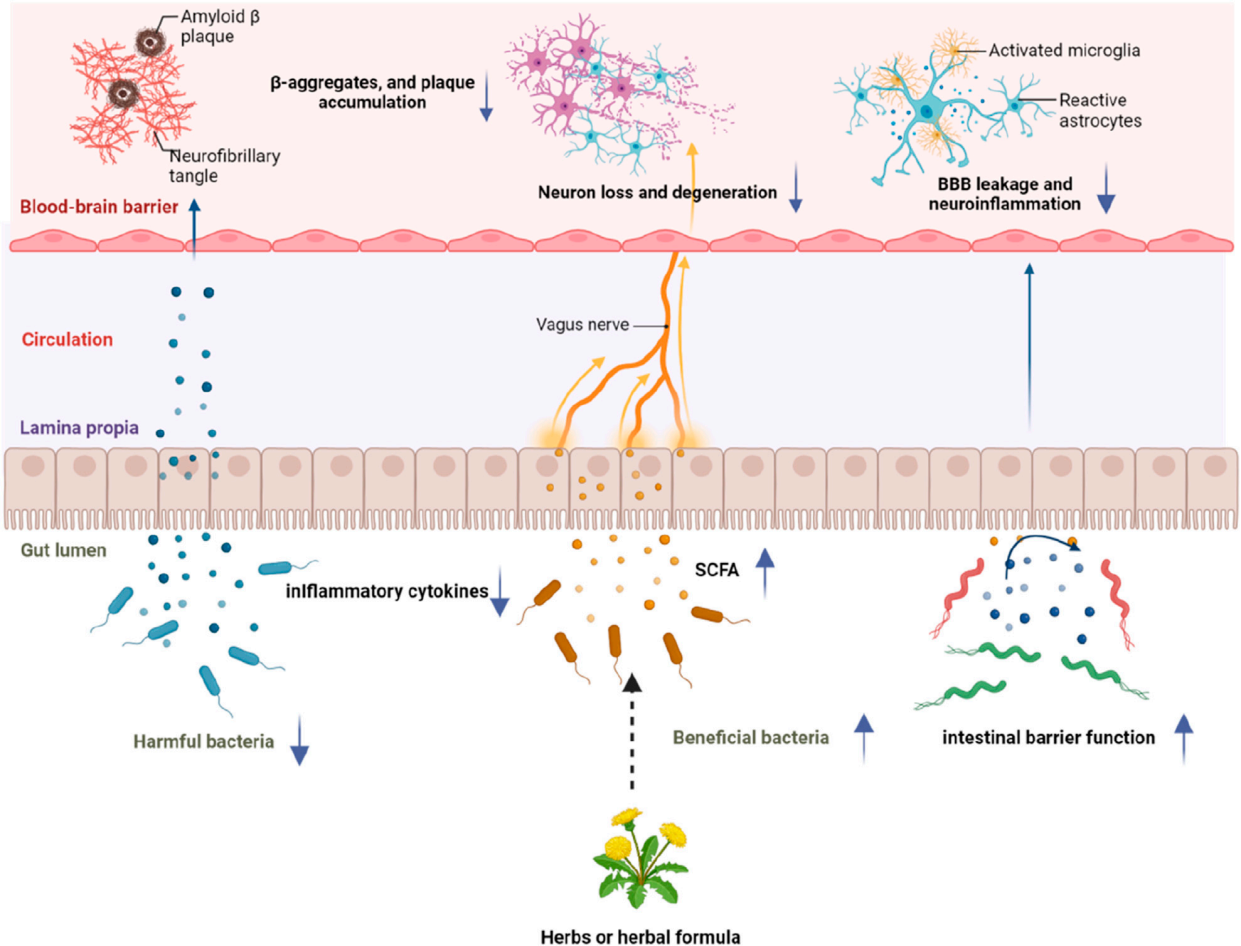
A schematic diagram of the influence of herbs or herbal formulae on gut microbiota and subsequent effects on AD progression through the microbiota-gut-brain axis. Ingredients of herbs or herbal formulae could modulate the composition of the gut microbiota by increasing the abundance of beneficial bacteria or decreasing the abundance of harmful bacteria, increasing the level of SCFA, decreasing the level of inflammatory cytokines, as well as improving the function of the intestinal barrier. These changes could subsequently alleviate blood-brain barrier alterations, β-aggregates, plaque accumulation, neuronal loss and neuroinflammation through the microbiota-gut-brain axis, and consequently slow down the progression of AD.

We reviewed commonly used herbs or herbal formulae for AD and their neuroprotective mechanisms, which were previously evaluated. Meanwhile, the effects of some herbs or herbal formulae on regulating gut microbiota were studied and the importance of herbs or herbal formulae in protecting against AD through modulating gut microbiota was elucidated. However, some herbs or herbal formulae have not been studied on their possible associations with gut microbiota, which may be the next step to further explore their neuroprotective mechanisms against AD in future studies. We also reviewed some herbs which have been shown to regulate gut microbiota. Specific gut microbesregulated by different herbal formulae, herbal extracts or herbs are shown in Table 5. Based on connections of gut microbiota with AD, herbal components may have secondary effects on the brain by affecting gut microbiota through regulating the microbiota-gut-brain axis, thereby influencing cognitive functions of the host. Herbal formulae or extracts of herbs modulate the composition of the gut microbiota by increasing the abundance of beneficial bacteria or decreasing the abundance of harmful bacteria, leading to an increased level of SCFA or decreased level of inflammatory cytokines. This further brings benefits to the function of the local intestinal barrier and the distal central nervous system, subsequently alleviating blood-brain barrier alterations, β-aggregates, plaque accumulation, neuronal loss and neuroinflammation through the microbiota-gut-brain axis, and consequently slow down the progression of AD. The impact of herbs or herbal formulae on gut microbiota and their subsequent effect on AD progression is shown in Figure 5.

**TABLE 5:** The specific affected gut microbes by different herbal formulae, herbal extracts or herbs.

Herbal formula	Upregulated bacteria	Downregulated bacteria
Jiedu-Yizhi formula	Lachnospiraceae↑,Ruminococcaceae↑, Actinobacteria↑	Alistipes↓,Muribaculaceae↓
Bushen-Huatan-Yizhi formula	*Proteus*↑,Actinomycetes↑, *Clostridium*↑, Lactococcus↑	-
Huanglian Jiedu decoction	Proteobacteria ↑, Prevotellaceae↑ Lactobacillaceae↑, Peptococcaceae↑	Firmicutes↓, Bacteroidales S24-7_group↓, Lachnospiraceae ↓, Porphyromonadaceae ↓
SuanZaoRen Decoction	*Firmicutes* phylum members↑	Bacteroidetes ↓
Naoxintong capsule	Ratio of the Firmicutes to Bacteroidetes ↑	-
Kai-Xin-San	Bifidobacterium ↑, *Helicobacter*↑	-
GuanXinNing Tablet	Akkermansia↑ dgA-11_gut_group↑	Firmicutes/Bacteroidetes ratio ↓
Qisheng Wan formula	*Lactobacillus* ↑	Alistipes ↓, Lachnospiraceae NK4A136 ↓
WangShiBoChiWan	*Lactobacillus*↑	*Bacteroides* fragillis ↓
Wumei decoction or pills	Allobaculum ↑, *Bacteroides* ↑	Ileibacterium↓
Extracts from herbs or herbs		
Cordycepin	Firmicutes/Bacteroidetes↑	-
ECa 233	-	*Staphylococcus*↓
OMO	*Lactobacillus*↑, Allobaculum↑ Lactobacillaceae↑	-
GEP-1	Akkermansia muciniphila ↑, Lacticaseibacillus paracasei ↑	-
Gastrodin	Firmicutes ↑	Cyanobacteria↓, Proteobacteria↓ Deferribaceters↓
Ponicidin	Allobaculum ↑, *Lactobacillus* ↑ Ruminococcus ↑	
6-gingerol	Bacteroidetes ↑	Firmicutes↓
WMP	*Lactobacillus* animalis ↑	*Fusobacterium* varium↓ *Bacteroides* vulgatus↓
GPWE and GP-IV	Butyricimonas↑, *Lactobacillus* ↑	Bilophila↓, Blautia ↓, Dorea ↓
Andrographolide	f__Lachnospiraceae_ Unclassified↑ Lachnospiraceae_ NK4A136_group ↑ Ruminococcaceae_ UCG-014↑	
Triptolide	Firmicutes↑	Bacteroidetes↓, *Bacteroides* ↓, Lachnospiraceae↓
PCA	Roseburia ↑, Desulfovibrio ↑	Prevotella 9↓, Prevotella 2↓, Runminococcus torques, Holdemanella↓
PLS	Firmicutes↑, *Lactobacillus* ↑	Proteobacteria↓, Escherichia-Shigella↓, *Enterococcus*↓
SAL	Alloprevotella ↑, Parasutterella ↑	Prevotellaceae ↓, Ruminiclostridium ↓
DJP	Prevotella↑, Akkermansia↑	*Escherichia coli*↓
RS	*Lactobacillus* ↑	
LB	Porphyromonadaceae↑, *Lactobacillus*↑ *Escherichia*↑	

Cordycepin: extracted from Cordyceps militaris; ECa: extracted from *Centella asiatica*; OMO: Oligosaccharides from Morinda officinalis; GEP-1: a polysaccharide from Gastrodia elata; Gastrodin: an active ingredient from Gastrodia elata; Ponicidin: a diterpenoid from Rabdosia rubescens; 6-gingerol: a polyphenol from Zingiber officinale Roscoe; WMP: extracts from walnut meal; GPWE: Gynostemma pentaphyllum water extract; Andrographolide: a compound isolated from Andrographis paniculata; Triptolide: extracted from Tripterygium Wilfordii Hook F; PCA: Protocatechuic acid, from Eucommia ulmoides Oliver; PLS: P. lobata starch, from Pueraria lobata (Willd.) Ohwi; SAL: Salidroside, a phenylpropanoid from Rhodiola rosea L.; DJP: rich-polyphenols extract of D. loddigesii; RS: Radix Scrophulariae; LB: Lilium brownii F. E. Brown.

## 7 Conclusion

Herbs or extracts from herbs are able to regulate the composition of the gut microbiota and they may be beneficial in protecting against AD through multiple pathogenic processes, so it is more attractive to find multipotent agents that can hit more than one target and exert better AD-preventing effects. Herbs, as naturally existing agents containing multipotent anti-AD compounds, are attracting much attention from the field.

Emerging evidence supports the use of herbal medicine for the treatment of AD, however, there is still a lack of direct evidence on the effect of herbal medicine on the composition of the gut microbiota in AD. Some herbal formulae or individual herbs have already been commonly used for treating age-related dementia. Therefore, medicinal herbs capable of modulating gut microbiota may become potential therapeutic candidates for AD.

A diversity of bioactive compounds from herbal medicines are mainly responsible for the anti-AD effects, such as flavonoids, alkaloids, Rosmarinic acid, xanthones (Gong et al., 2020). As they are capable of regulating the balance of the gut microbiota, it is postulated that these herbal medicines may improve AD by targeting the composition of gut microbiota. Though there is evidence supporting the use of herbal medicines in the prevention and treatment of AD, clinical evidence of their effectiveness, especially from phase II and III clinical trials, is still in its infancy. The universal use of herbs in the management of AD patients needs to be evaluated in clinical trials like FDA approved drugs.

## 8 Limitations and future perspectives

Evidence supports the beneficial effect of herbal medicine for the treatment of AD, but some obstacles can not be neglected for the application of herbal medicine. As the compounds in herbs are complex, the mechanisms of action, specific features of metabolism of some compounds are still not elucidated. Thus, metabolomics may be a promising method to better explore active ingredients and to clarify mechanisms and metabolic pathways of herbal medicine from a molecule metabolism level (Ren et al., 2023). Besides, herbs and herbal ingredients usually were transformed into metabolites with a higher bioavailability and activities by the gut microbiota after entering into the gut. To better explore the activities and mechanisms of metabolites of herbal ingredients, a novel strategy is proposed which involves spectrum-effect relationship analysis, network pharmacology, metabolomics analysis and molecular docking. To be specific, the herb and bioactive metabolites were screened through spectrum-effect relationship analysis firstly. Network pharmacology and metabolomics analysis were utilized to identify the upstream key targets of the herb and its metabolites as well as the downstream endogenous metabolites secondly. Lastly, the active forms of herbs were further confirmed by molecular docking (Zhong et al., 2023). However, as the biotransformation of the herbal ingredients is also very complicated, it remains a challenge to confirm the activities and mechanisms of the metabolites of herbal ingredients.

Compared with synthetic drugs, herbal plants and bioactive compounds are superior with regard to safety, however, the safety aspect of medicinal plants and bioactive candidates needs to be assessed through animal studies and clinical trials to develop evidence-based medicines for AD.
